# Humoral immune responses primed by the alteration of gut microbiota were associated with galactose-deficient IgA1 production in IgA nephropathy

**DOI:** 10.3389/fimmu.2024.1415026

**Published:** 2024-07-22

**Authors:** Li Gao, Huixian Li, Xiaoling Liu, Haiyun Li, Peiqi Li, Wanhong Lu, Xinfang Xie, Jicheng Lv, Jing Jin

**Affiliations:** ^1^ Department of Nephrology, The First Affiliated Hospital of Xi’an Jiaotong University, Xi’an, China; ^2^ Department of Cardiology, The First Affiliated Hospital of Xi’an Jiaotong University, Xi’an, China; ^3^ MOE Key Laboratory of Cell Activities and Stress Adaptations, School of Life Science, Lanzhou University, Lanzhou, China; ^4^ MOE Key Laboratory of Environment and Genes Related to Diseases, School of Basic Medical Sciences, Xi’an Jiaotong University, Xi’an, China; ^5^ Renal Division, Peking University First Hospital; Peking University Institute of Nephrology, Key Laboratory of Renal Disease, Ministry of Health of China, Key Laboratory of Chronic Kidney Disease Prevention and Treatment (Peking University), Ministry of Education, Beijing, China; ^6^ Department of Medicine-Nephrology and Hypertension, Feinberg Cardiovascular and Renal Research Institute, Northwestern University Feinberg School of Medicine, Chicago, IL, United States

**Keywords:** IgA nephropathy, galactose-deficient IgA1, Escherichia-Shigella, mucosal immunity, IgA-protease, commensal bacteria

## Abstract

**Introduction:**

Galactose-deficient IgA1 (GdIgA1) is critical in the formation of immunodeposits in IgA nephropathy (IgAN), whereas the origin of GdIgA1 is unknown. We focused on the immune response to fecal microbiota in patients with IgAN.

**Methods:**

By running 16S ribosomal RNA gene sequencing, we compared IgAN samples to the control samples from household-matched or non-related individuals. Levels of plasma GdIgA1 and poly-IgA complexes were measured, and candidate microbes that can either incite IgA-directed antibody response or degrade IgA through specific IgA protease activities were identified.

**Results:**

The IgAN group showed a distinct composition of fecal microbiota as compared to healthy controls. Particularly, high abundance of Escherichia-Shigella was associated with the disease group based on analyses using receiver operating characteristic (area under curve, 0.837; 95% CI, 0.738–0.914), principle coordinates, and the linear discriminant analysis effect size algorithm (linear discriminant analysis score, 4.56; *p* < 0.001). Accordingly, the bacterial levels directly correlated with high titers of plasma GdIgA1(*r* = 0.36, *p* < 0.001), and patients had higher IgA1 against stx2(2.88 ± 0.46 IU/mL vs. 1.34 ± 0.35 IU/mL, *p* = 0.03), the main antigen of Escherichia-Shigella. Conversely, the healthy controls showed relatively higher abundance of the commensal bacteria that produce IgA-degrading proteases. Particularly, the abundance of some intestinal bacteria expressing IgA proteases showed an inverse correlation with the levels of plasma GdIgA1 in IgAN.

**Conclusion:**

Our data suggest that mucosal IgA production, including those of GdIgA1, is potentially linked to the humoral response to gut Escherichia-Shigella as one of the sources of plasma GdIgA1. Conversely, the IgA protease-producing microbiota in the gut are suppressed in patients with IgAN.

## Introduction

IgA nephropathy (IgAN) is the most common primary glomerulonephritis worldwide and a leading cause of end-stage kidney disease (1, 2). Deposition of predominant IgA1 in the glomerular mesangial region is the diagnostic hallmark in kidney biopsy. At the molecular level, IgA1 with poorly glycosylated hinge segment of the heavy chain [referred to as galactose-deficient IgA1 (GdIgA1)] is particularly prone to deposition and is believed to have a major role in the pathogenesis of IgAN (3, 4). However, the underlying mechanisms for the production of GdIgA1 are incompletely understood. Evidence suggests that GdIgA1 is systemically produced, and there is a high recurrence rate of IgAN in the graft kidney following transplantation (5, 6). Accordingly, when kidney grafts from IgAN donors are transplanted to non-IgAN recipients, the original IgA deposits in the donor kidney may gradually disappear over time.

Regarding the site of GdIgA1-producing plasma cells, the significant role of aberrant mucosal immune responses in the pathogenesis of IgAN has been highlighted. In the absence of timely biopsy data, gross hematuria is often the first attack symptom of IgAN 12–24 h following mucosal infection (7). Frequent mucosal infection is also a risk factor of IgAN flare and progression, and topical corticosteroid budesonide for treating inflammatory conditions of the ileal gut-associated lymphoid system is a targeted therapy for IgAN (8). Additionally, genome-wide association studies identified susceptibility genes for IgAN with functions in intestinal immunity (9). Numerous studies demonstrated that mucosal-derived antigens stimulate the differentiation of B cells into IgA-secreting plasma cells, through T-cell–independent or T-cell–dependent pathways, inducing an immunoglobulin class switching from IgG/IgM to IgA (10, 11). Studies also showed that the mucosal plasma B cells tend to secrete GdIgA1 (12), and limited literature supported the hypothesis that the mucosae-derived GdIgA1+ plasma cells mis-home to the bone marrow during lymphocyte trafficking (11, 13) or retrotranscytosis of mucosal GdIgA1 across human epithelium to circulating system (14, 15). Meanwhile, long-lived plasma cells can also travel between mucosal sites and the bone marrow (16, 17).

Gut microbiotas are known to play an important role in improving the production of IgA. Recent studies have focused on the alterations of the intestinal microbiota and intestinal mucosal hyperresponsiveness in association with high GdIgA1 production in IgAN (14, 18–21). In a genetic model with the overexpression of B-cell activation factor, McCarthy and colleagues showed that high levels of IgA antibody response to the intestinal bacteria can lead to mouse phenotypes reminiscent of IgAN (22). Challenged by microbes in conventional environment, humanized IgA1 transgenic mice had more IgA deposition than those in a specific pathogen–free environment (23). Furthermore, fecal transplantation from patients with IgAN to humanized IgA transgenic mice can also cause IgAN-like phenotypes (24), and depleting the intestinal microbiota with antibiotics could make the disease curable in a humanized mouse model of IgAN (25). However, very few studies elucidated the precise gut microbiota and the conditions that can incite the production of GdIgA1 to cause IgAN. In this study, we analyzed the composition of fecal microbiome in patients with IgAN and discovered an imbalance between GdIgA1-stimulating and mucosal IgA–degrading bacterial activities in disease.

## Materials and methods

### Study subjects

A total of 146 participants, including 77 cases with IgAN, 22 household-matched healthy controls (HM-HCs) for 22 corresponding patients with IgAN, and 47 non-related healthy controls (HCs), were recruited from the First Affiliated Hospital of Xi’an Jiaotong University. Patients with IgAN were all diagnosed by renal biopsy with predominance of IgA deposits in the glomerular mesangium. Patients younger than 18 years old, with gastrointestinal diseases, having secondary IgAN, or having other autoimmune diseases or diabetes were excluded. Other exclusion criteria include the use of probiotics, antibiotics, glucocorticoid, or other immunosuppressants within 2 months. Twenty-two HM-HCs were selected for addressing confounding factors such as dietary habits and environmental factors. Participants between groups were matched for age and sex. Patients’ clinical characteristics, including age, sex, blood pressure, 24-h urine protein excretion, serum albumin, creatinine, estimated glomerular filtration rate (eGFR), and pathologic scores of Oxford Classification for IgAN were documented. This study was performed in adherence to the Declaration of Helsinki and was approved by the medical ethics committee at the First Affiliated Hospital of Xi’an Jiaotong University. Plasma and stool samples from all patients with IgAN were collected at the time of renal biopsy, and all samples were stored at −80°C within 2 h of collection.

### Detection of plasma IgA1, GdIgA1, IgG anti-glycan antibodies, poly-IgA complexes, and anti-stx2 IgA1 by ELISA

Plasma IgA1 was measured using standard enzyme-linked immunosorbent assay (ELISA) as previously described (26). Plasma GdIgA1 levels were quantified using the GdIgA1 assay kit (IBL, Naka, Japan) following the manufacturer’s standard protocol.

Method for detecting anti-glycan antibodies against GdIgA1 was described previously (26). Briefly, IgA1 F(ab)2 plus the hinge region [F(ab)2-HR] was isolated following IgA protease digestion of purified IgA1 from plasma. Following protein-L column purification of the F(ab)2 fragment, 5 μg/mL was used to coat ELISA plates as antigen. After blocking with 1% bovine serum albumin (BSA) in phosphate-buffered saline (PBS) for 1 h at 37°C, 100 times diluted plasma samples and standards were added to the assigned wells and incubated for 1 h at 37°C. Finally, alkaline phosphatase–conjugated goat anti-human IgG monoclonal antibody (Sigma, United States) was used for detection.

The method of using recombinant CD89 (rCD89) to capture poly-IgA complexes in the plasma was reported previously (27). Briefly, rCD89 (5 μg/mL; SinoBiological, China) as a capturing agent was used to coat the ELISA plate, after blocking with 1% BSA/PBS buffer for 2 h at 37°C, and diluted plasma samples (1:1,000) were added and incubated for 3 h at 37°C. Then, horseradish peroxidase (HRP)-labeled mouse anti-human IgA mAb (Abcam, Cambridge, United Kingdom) diluted 1:1,000 in blocking buffer was added to the wells for 1 h at 37°C. Lastly, results were developed using 3,39,5,59-tetramethylbenzidine liquid substrate, and the reactions were stopped with the addition of 1 M sulfuric acid.

For detecting anti-Shiga toxin 2 (stx2) antibody/IgA1 in plasma, recombinant stx2 (5 μg/mL; SinoBiological, China) was used as an antigen to coat the ELISA plate. BSA was used as the control antigen. After blocking, all plasma samples at 1:50 dilution and one sample in a two-fold dilution series as the standards were added to the wells. After 1-h incubation at 37°C and subsequent washing steps, HRP-conjugated mouse anti-human IgA1 antibody (1:2,000 dilution, ThermoFisher) was added to the wells for 1 h at 37°C. The results for total CD89-captured poly-IgA complexes and anti-stx2 IgA1 were expressed as units per milliliter.

### 16S ribosomal RNA gene sequencing and data preprocessing

Microbial DNA was extracted from the fecal samples using a HiPure Soil DNA-extraction kit (Magen, Guangzhou, China) following the manufacturer’s standard protocol. The V3-V4 hypervariable region of the 16S ribosomal RNA (rRNA) genes was amplified by PCR using primer pair 341F (5′-CCTACGGGNGGCWGCAG-3′) and 806R (5′-GGACTACHVGGGTATCTAAT-3′). Then, the amplified 16S rRNA PCR fragments were sequenced using Illumina Nova SP (PE250) at Gene Denovo Biotechnology Co., Ltd. (Guangzhou, China). To further improve read quality, raw reads were filtered on the basis of a set of rules (operated using FASTP, version 0.18.0) (28). The clean tags were clustered into operational taxonomic units (OTUs) of ≥97% similarity using UPARSE (29) (version 9.2.64). Bioinformatic analyses, including alpha diversity analysis, beta diversity analysis, community composition analysis, indicator species analysis, and function prediction were all performed using Omicsmart, a real-time interactive online platform for data analysis (http://www.omicsmart.com). Additional methods on 16S rRNA gene sequencing are in **Supplementary Materials**.

### Statistical analysis

Normally distributed and non-normally distributed quantitative parameters were expressed as means ± standard deviation, medians, and interquartile ranges (IQRs), respectively. Statistical differences between two groups in normal distribution were analyzed using a two-tailed Student *t*-test. Statistical methods for bioinformatic analyses of the 16S rRNA gene sequencing data were shown in **Supplementary Materials**. Levels of plasma GdIgA1, anti-glycan antibodies, rCD89-capturing IgA complexes, and anti-stx2 IgA1 between the groups were plotted using Prism software (GraphPad Software, La Jolla, CA). A two-tailed *p*-value of <0.05 was considered statistically significant. All other statistical analyses were performed using SPSS version 20.0.

## Results

### General characterization of the IgAN cohort

**Figure 1:**
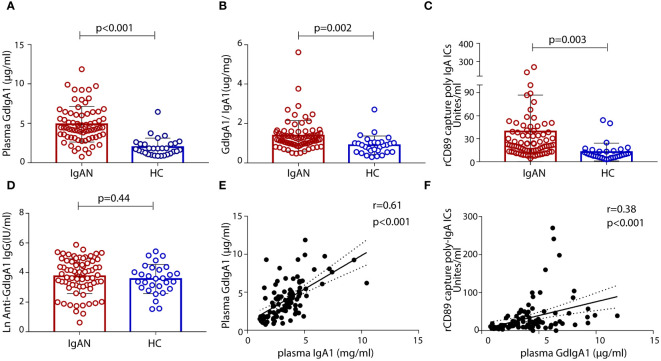
Levels of plasma galactose-deficient IgA1 (GdIgA1), anti-glycan antibodies, and poly-IgA1 complexes in patients of IgA nephropathy and healthy controls. Patients with IgAN had significantly higher levels of plasma GdIgA1 **(A)**, GdIgA1/IgA1 **(B)**, and poly-IgA1 **(C)** than healthy participants. Levels of plasma autoantibodies against GdIgA1 were comparable between the two groups **(D)**. Plasma IgA1 and poly-IgA1 levels were positively correlated with circulating GdIgA1 levels (*r* = 0.61, *p* < 0.001 for plasma IgA1 and GdgA1 and *r* = 0.38, *p* < 0.001 for poly-IgA1 and GdIgA1, respectively, **E**, **F**).

We included 77 patients with biopsy-confirmed IgAN, 22 household-matched healthy subjects, and an additional 47 non-related healthy volunteers as controls in this study. A summary of the baseline characteristics of the patients with IgAN is presented in **Table 1**. Sex and age were balanced between the patient and the control groups. Most patients with IgAN had preserved renal function, with their levels of eGFR at 84.8 ± 73.0 mL/min/1.73 m^2^ and proteinuria at 2.1 ± 1.9 g/day. Based on the Oxford Classification of IgAN lesions, 90.9% patients had mesangial proliferation (M1), 76.6% patients had segmental sclerosis (S1) lesions, 39% patients had interstitial fibrosis/tubular atrophy (T1/T2) lesions, and 39% patients had glomerular crescents (C). Serological measurements of plasma GdIgA1, plasma autoantibodies against GdIgA1, and plasma poly-IgA1 complexes were also performed. As expected, the IgAN group had significantly higher levels of GdIgA1, GdIgA1/IgA1, and poly-IgA1 complexes than the control groups (4.89 ± 0.27 μg/mL vs. 1.97 ± 0.2 μg/mL, 1.38 ± 0.09 μg/mg vs. 0.89 ± 0.09 μg/mg, and 39.3 ± 5.3 vs. 12.6 ± 2.1 U/mL, respectively; *p* < 0.05; **Figures 1A–C**). In contrast, the titers of anti-GdIgA1 IgG autoantibodies were comparable between IgAN and controls (**Figure 1D**). Overall, there were positive correlations between plasma GdIgA1 and total IgA1 (*r* = 0.61, *p* < 0.001; **Figure 1E**) and between plasma GdIgA1 and poly-IgA1 complexes (*r* = 0.38, *p* < 0.001; **Figure 1F**), supporting the notion of GdIgA1 being pathogenic and prone to aggregation (30).

**Table 1:** Baseline characteristics of included patients with IgAN and healthy controls.

Baseline characteristics	IgAN (n = 77)	Non-related healthy controls (n = 47)	Household-matched healthy controls (n = 22)
**Age (years old)**	35.4 ± 11.8	33.5 ± 10.9	34.6 ± 8.2
**Sex (male %)**	57.10%	54.35%	53.62%
**SBP (mmHg)**	130 ± 20	120 ± 14	122 ± 16
**DBP (mmHg)**	88 ± 14	76 ± 11	79 ± 10
**Serum albumin (g/L)**	35.5 ± 8.5	38.5 ± 3.8	37.7 ± 4.3
**Serum creatinine (μmol/L)**	107.7 ± 73.1	68.5 ± 21.1	72.2 ± 25.3
**eGFR (mL/1.73m^2^)**	84.8 ± 73.0	119.8 ± 22	109.8 ± 25
**Hemoglobin (g/L)**	135 ± 26	138 ± 23	136 ± 27
**Total urinary protein (g/day)**	2.1 ± 1.9	─	─
**CKD stage 3–5 (%)**	29.9	─	─
**M1 (%)**	90.9	─	─
**E1 (%)**	18.2	─	─
**S1 (%)**	76.6	─	─
**T1/2 (%)**	39	─	─
**C1/2 (%)**	39	─	─

SBP, systolic blood pressure; DBP, diastolic blood pressure; eGFR, estimated glomerular filtration rate; CKD, chronic kidney disease; M, mesangial proliferation; S, segmental sclerosis lesions; T, interstitial fibrosis/tubular atrophy; C, glomerular crescents.

### 16S rRNA gene sequencing of fecal collections identified gut microbial genera associated with IgAN

By performing 16S rRNA gene sequencing, we compared the fecal microbiota of patients with IgAN versus household-matched or non-related HCs. Alpha diversity analyses of Sob, Chao1, Abundance-based Coverage Estimator (ACE), Shannon, Simpson, and rank abundance all showed evenness among the groups (**Supplementary Figures S1A–F**), suggesting that, at the phylum and the genus levels, there were no major dysbiosis of gut microbiota in IgAN. At the genus level, patients with IgAN had 63 unique genera that were largely absent in household-matched and non-related healthy volunteers (**Supplementary Figure S1G**). The compositions of the genera are shown in **Supplementary Figure S1H**. The main genera such as Bacteroides, Faecalibacterium, Parabactrium, Megamonas, Prevotella_9, Escherichia-Shigella, Dialster, Lachnoclostridium, and phascolarctobacterium were identified in all three groups (further details in **Supplementary Table S1**).

**Figure 2:**
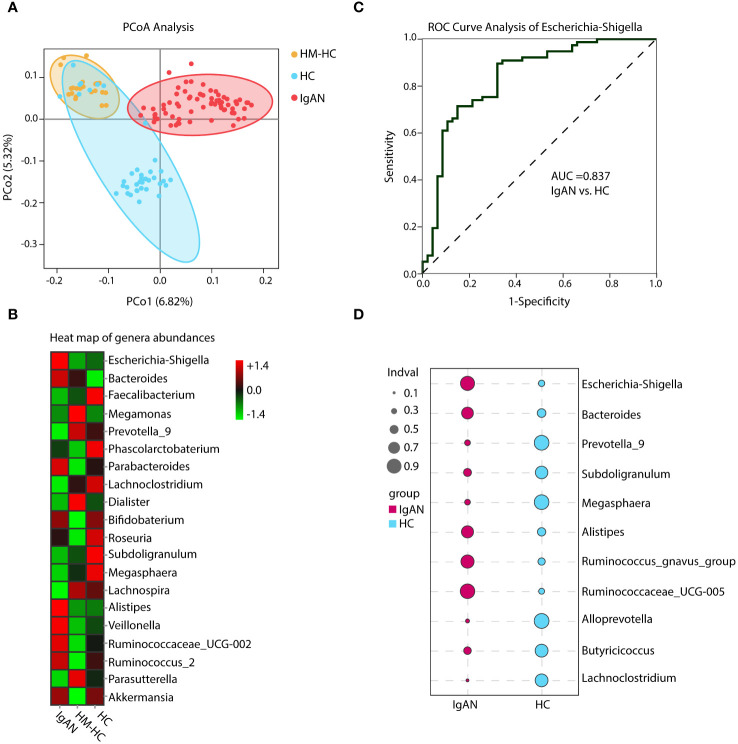
Principal coordinate analysis (PCoA), heat map of genera abundances, ROC curve, and indicator analysis of the genera. PCoA analysis and heat map based on the composition of the genera showed substantially different distributions in the IgAN group as compared to the control groups **(A, B)**. The IgAN group had higher relative abundance of Escherichia-Shigella than the healthy control groups. ROC analysis demonstrated that Escherichia-Shigella had the best AUC value among all genera to differentiate patients with IgAN from healthy controls **(C)**. Patients with IgAN also had significantly higher indicator values as calculated based on abundance and frequency of Escherichia-Shigella than healthy control participants (0.77 vs. 0.22, p = 0.002). Other genera such as Bacteroides, Prevotella_9, and Subdoligranulum with significantly different indicator values between groups could also be considered in the group’s discrimination **(D)**. HM-HC, household-matched healthy control; HC, healthy control; IgAN, IgA nephropathy.

By performing beta diversity analysis of Jaccard distance with Welch’s t-test, we detected an imbalance in the microbiome in the IgAN group as compared to the HCs (*p* < 0.001). These differences were evident in the principal coordinate analysis (PCoA) using unweighted unifrac distances in the OTU level (**Figure 2A**). The relative abundance of selected genera across the three groups is depicted by heat maps (**Figure 2B**), in which the IgAN group shows a significant enrichment of Escherichia-Shigella, Bacteroides, and Alistipes. Meanwhile, the abundance of Faecalibacterium, Prevotella_9, and Lachnoclostridium genera was lower in the IgAN group (**Figure 2B**; **Supplementary Table S1**). Importantly, similar differences of the genera were also evident when we individually compared IgAN subjects with their household-matched family members (**Supplementary Figure S2A**), suggesting that the observed enrichments of these genera in IgAN could possibly link to the disease.

### Enrichment of Escherichia-Shigella as an indicator genus in IgAN

**Figure 3:**
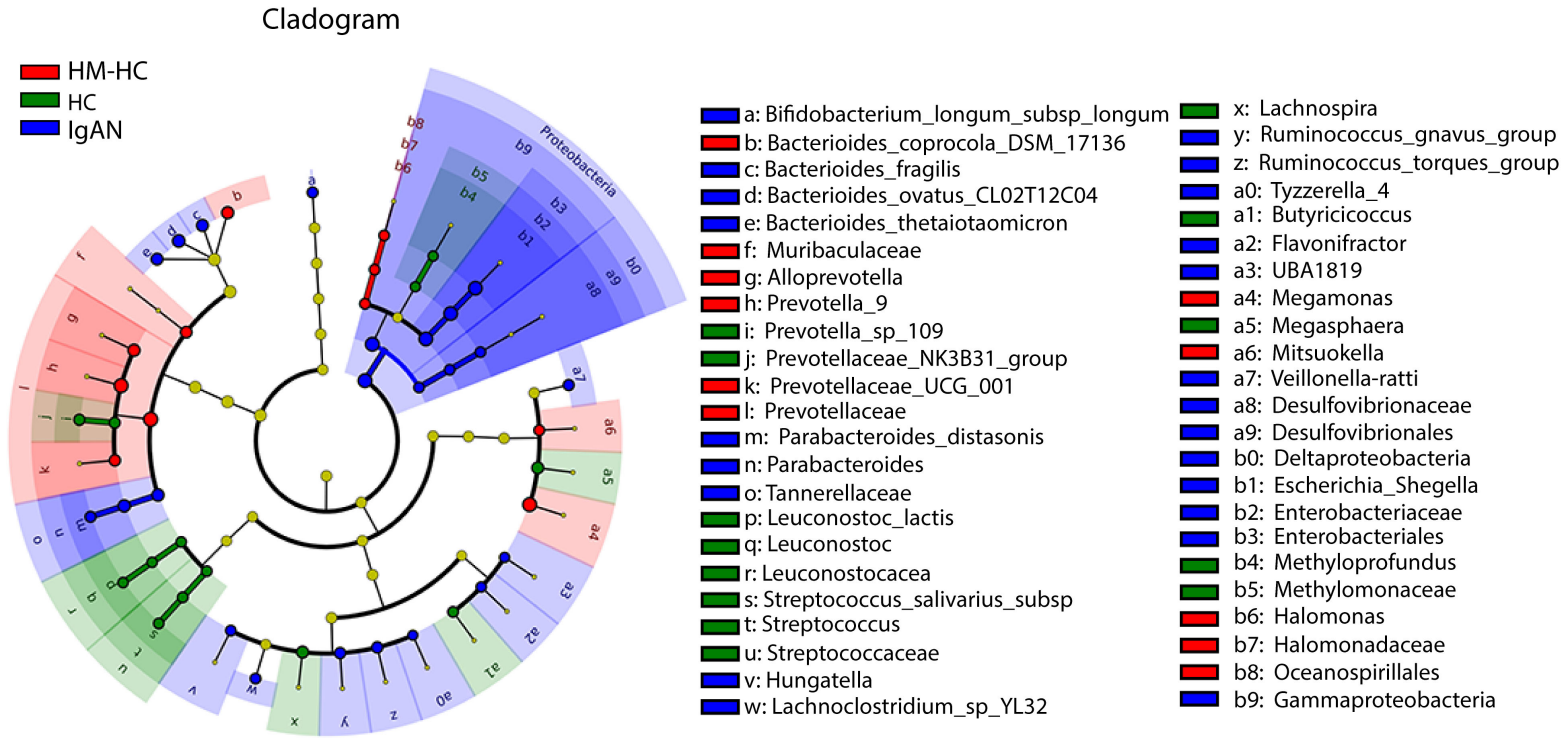
Characterization of gut microbiota differences between patients with IgAN and healthy controls by using LEfSe analysis and LAD. Linear discriminant analysis (LDA) and effect size (LEfSe) analysis for high-dimensional biomarker discovery and explanation were conducted. Significantly enriched bacterial taxa were identified in patients with IgAN (bule) with threshold of >3.0. Among them, Enterobacteriaceae, Enterobacteriales, and Escherichia-Shigella with higher LDA values were highly enriched in IgAN, HM-HC, and HC. HM-HC, household-matched healthy control; HC, healthy control; IgAN, IgA nephropathy.

Further analysis of the results using receiver operating characteristic curve (ROC) showed that Escherichia-Shigella had the highest area under curve (AUC) value of 0.837 (95% CI, 0.738–0.914; additional details in **Supplementary Table S2**) among all identified genera in association with IgAN (**Figure 2C**). Escherichia-Shigella also had the higher indicator value of 0.77 for IgAN as a group versus 0.23 for HCs shown in the bubble chart (*p* = 0.002, **Figure 2D**), supporting it having a high probability as an indicator genus between groups. Again, when patients were individual compared to their household-matched counterparts, Escherichia-Shigella abundance was the best marker to distinguish the pairs (**Supplementary Figures S2B–E**), further implicating the genus being linked to IgAN. Additionally, we performed linear discriminant analysis (LDA) by running the LEfSe algorithm to compare patients with IgAN to HCs (**Figure 3** and **Supplementary Table S3**; threshold of LDA score of >3, *p* < 0.05). The results also showed high abundance of Escherichia-Shigella associated with IgAN (LDA score: 4.56, *p* < 0.001; **Supplementary Figure S3**), in contrast to the enrichment of Prevotellaceae, Megasphaera, and Prevotella_9 in controls (**Figure 3**; **Supplementary Figure S3**; **Supplementary Table S3**). Once more, individually paired analysis of IgAN versus their corresponding household controls in LEfSe also identified Escherichia-Shigella as one of the best indicators for the disease (**Supplementary Figure S4**; **Supplementary Table S4**). Collectively, these results supported fecal Escherichia-Shigella as a marker in differentiating patients with IgAN from HCs, which prompted us to examine its antigenicity.

### Escherichia-Shigella correlated with high plasma GdIgA1 levels, and patients with IgAN developed antibodies against Escherichia-Shigella antigen stx2

**Figure 4:**
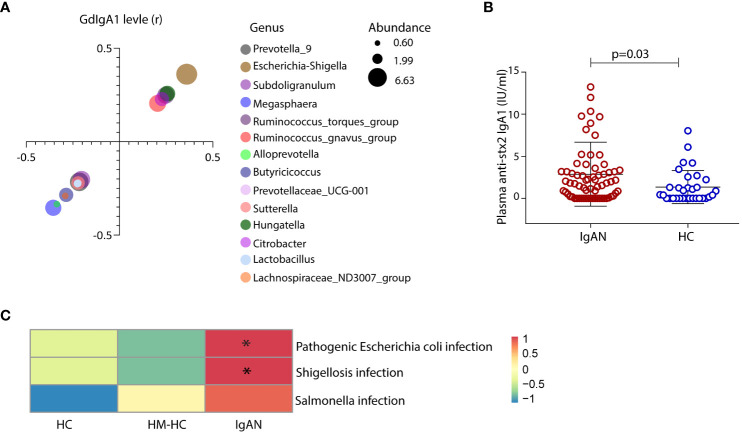
Exposure of gut Escherichia-Shigella was associated with the levels of circulating GdIgA1 and plasma IgA1 specific against the bacteria was detected in IgAN. A variety of gut bacteria correlated with high plasma GdIgA1 levels in patients with IgAN (p < 0.05) **(A)**. At the top of the list, Escherichia-Shigella correlated strongly with plasma GdIgA1 levels (r = 0.36, p < 0.001). In contrast, Prevotella_9 Alloprevotella, Butyricicoccus, and Lachnospiraceae_ND3007_group were negatively correlated with plasma GdIgA1 levels. Patients with IgAN had elevated plasma IgA1 against the stx2 antigen of Escherichia-Shigella **(B)**. **(C)** Tax4fun functional analysis and KEGG pathway analysis of level 3 predicted higher proportions of pathogenic Escherichia-coli infection in the IgAN group than in the healthy controls (p = 0.003 for IgAN vs. HC and p < 0.001 for IgAN vs. HM-HC). HM-HC, household-matched healthy control; HC, healthy control; IgAN, IgA nephropathy; stx2, Shiga toxin 2. * means p<0.05 when compared to the other groups, respectively.

We sought to investigate whether gut mucosal–derived plasma B cells (11, 13) produce GdIgA1 in response to Escherichia-Shigella. Firstly, we compared plasma GdIgA1 levels to the relative abundance of selected fecal microbiotas. High abundance of Escherichia-Shigella, Hungatella, Ruminococcus gnavus, and Ruminococcus torques generally correlated with high GdIgA1 levels as seen in patients with IgAN (*p* < 0.05, **Figure 4A**), with Escherichia-Shigella ranked the highest (*r* = 0.36, *p* < 0.001). Meanwhile, the abundance of Magosphaera and Prevotella_9, Alloprevotella, Butyricicoccus, Succiniclasticum, and Lachnospiraceae_ND3007_group inversely correlated with plasma GdIgA1 levels. To test whether Escherichia-Shigella could directly elicit antibody responses, we coated plates with the main antigen of Escherichia-Shigella, stx2, for detecting antimicrobial antibodies in the hosts, particularly those of the IgA1 type. Interestingly, anti-stx2 IgA1 is more frequently detected in patients with IgAN than in HCs (2.88 ± 0.46 IU/mL vs. 1.34 ± 0.35 IU/mL, *p* = 0.03; **Figure 4B**). Furthermore, functional evaluation of gut microbial by Tax4fun in conjunction with Kyoto Encyclopedia of Genes and Genomes (KEGG) pathway analyses of level 3 predicted that higher proportions of infections by Escherichia coli and Shigella are strongly associated with the IgAN group than with the controls (**Figure 4C**; *p* = 0.003 for IgAN vs. HC and *p* < 0.001 for IgAN vs. HM-HC; **Supplementary Figure S5**), whereas the Salmonella infection rate was comparable among the groups. Collectively, the 16S rRNA data suggest a potentially causal relationship between IgA1 responses to gut Escherichia-Shigella and the development of IgAN.

### IgAN is associated with the suppression of intestinal flora that produce IgA-degrading proteases

**Figure 5:**
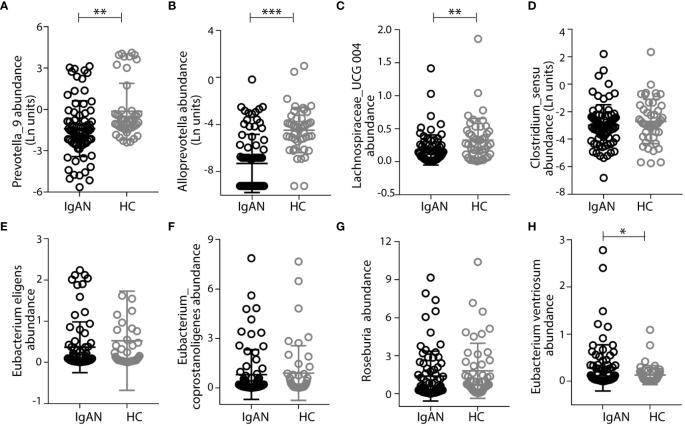
Patients with IgAN had low abundance of intestinal commensal flora that express the M64-class IgA proteases. Prevotalla_9, Alloprevotalla, and Lachnospiraceae_UCG 004 were with significantly lower abundance in IgAN than in healthy control (HC) **(A–C)**. Other bacteria, including Clostridium_sensu, Eubacterium eligens, Eubacterium Coprostanoligenes, and Roseburia, that have genes encoding M64-class IgA proteases had lower abundances in IgAN than in healthy controls **(D–G)**, with the only exception of Eubacterium ventriosum **(H)**. Data were described as mean ± standard deviation in each group. IgAN, IgA nephropathy; HC, healthy control. *** p<0.001, **p<0.01, *P<0.05.

In addition to focusing on IgA1-stimulating bacterial activities, we also studied the gut microbiome that expresses IgA-specific bacterial proteases [referred to as IgA-P (31)]. We speculated that their collective activities in the intestine contribute to the natural catabolism of IgA, which may ultimately affect total IgA levels in circulation. Previously, we and others studied a commensal strain of Clostridium ramosum on its IgA-P known as AK183 (32). By extensively searching for AK183-like bacterial proteases based on sequence similarities, using an open-source database (33) (https://www.ebi.ac.uk/merops/), we enlisted >100 proteases that are generally known as M64-type peptidases (**Supplementary Figure S6**). Among those that the host species are traceable, we discovered that a majority of these species reside in the human intestinal and, therefore, likely constitute the microbial flora (shown as highlighted in **Supplementary Figure S6**). Notably, many of the host species with IgA M64 peptidases belong to Clostridium senus including Eubacterium ventriosum, Coprobacillus sp., Roseburia intestinalis, Lachnospiraceae, and Eubacterium eligens, among others (**Supplementary Figure S6**). Other intestinal microbiota with potential IgA-Ps activity belong to the genus of Prevotella spp. or Alloprevotella genera (**Supplementary Table S5**). To investigate their potential contribution in catabolizing IgA in the gastric intestinal tract to ultimately lower the overall IgA load in circulation, we compared the relative levels of Prevotella spp. and Alloprevotella in IgAN to HC by 16S rRNA gene sequencing. Although not all species of gut Prevotella spp. and Alloprevotella are IgA-P–producing bacteria, the Prevoltella spp. and Alloprevotella genera had significantly lower abundance in the IgAN group than that in HCs (**Figures 5A, B**). In addition, Prevoltella spp. and Alloprevotella abundances were both inversely correlated with plasma GdIgA1 levels (**Figure 4A**), suggesting their role in catabolizing IgA through M64 IgA-P activities. In keeping with our hypothesis that IgA catabolism through collective activities of IgA-Ps may contribute to lowering the overall IgA load, we discovered a general trend of reduced carriage of commensal clostridium bacteria with IgA-P activities, such as Lachnospiraceae_UCG 004, Clostridium_sensu, Eubacterium eligens, Eubacterium Coprostanoligenes, and Roseburia in patients with IgAN as compared to the HCs (**Figures 5C–G**), with the only exception of poorly detected Eubacterium ventriosum species (**Figure 5H**).

## Discussion

IgAN is a complex disease caused by the aberrant production of GdIgA1 as the main source of immunodeposits. Clinical evidence points to a connection between mucosal hyperimmunoreactivity and the kidney that had led to the concept of “gut-kidney” axis in IgAN (12, 34). As the mucosal lymphoid synthesizes the bulk of IgA in the body, studies have focused on the gut microbiota for their roles in stimulating antibody responses (35). We followed a 16S rRNA gene sequencing approach to characterize the fecal composition of the microbiome among patients with IgAN as compared to HCs. We were interested in identifying key bacterial taxa that are enriched in disease for inciting antigenicity. Meanwhile, previous studies indicated retrotranscytosis, or spillover, of mucosal IgA across human epithelium (36) involved in the pathogenesis of IgAN (14, 15). Our 16S rRNA results discovered the Escherichia-Shigella genera for being overpopulated in the gut, and a high level of IgA1 antibody response to their main bacterial antigen stx2 was elevated among patients with IgAN. These results are consistent with the observation of high circulating GdIgA1 levels being positively correlated with the abundance of Escherichia-Shigella (37). In addition to focusing on antigenic bacteria in the gut, we also characterized microflora that naturally express IgA-degrading proteases (IgA-P). To this end, we analyzed taxa with subgroups of gut bacteria known to express the M64 class of IgA proteases. Earlier work of our own (32) and information from Merops Database on peptidase classification (33) collectively showed M64 proteases being expressed by commensal bacteria. We recognized Clostridium and Prevoltella genera that express M64 have lower representations in the IgAN group than the healthy group. Therefore, it is plausible that dysbiosis that shifts the balance between IgA-stimulating Escherichia-Shigella and IgA-degrading bacteria such as Prevoltella may ultimately lead to high GdIgA1 levels in circulation as seen in IgAN.

Circulating GdIgA1 and its polymerization play a crucial role in the pathogenesis of IgAN (38). As the clinical onset of gross hematuria or an episode of heavy proteinuria in patients is often preceded with a mucosal infection, it is suggested that mucosal immunity may have a causal role in some cases of IgAN (7). By tracing the clonal plasma B-cell lineage, a strong relatedness between mucosal and systemic plasma cell pool was discovered, which is consistent with the notion of mucosal priming of IgA-producing plasma cells (39–41). This concept is further supported by the discoveries of susceptibility genes that regulate gut-associated lymphoid tissue (GALT) functions against gut pathogens (9, 10, 42). Accordingly, new synthetic corticosteroid Nefecon specifically formulated to suppress GALT immunity has shown renal protection in IgAN with reduced systemic toxicity (8). Our own study here is focused on the imbalance of gut microbiota taxa. One of our discoveries is the correlation between the abundance of Escherichia-Shigella genera and the individuals’ overall plasma GdIgA1 levels and the detection of anti–Escherichia-Shigella IgA antibodies in circulation. In this regard, our results provided new supporting evidence for the causal role of the gut-kidney axis in IgAN (**Supplementary Figure S7**).

We should also note that prior to our study, there have been several publications on fecal expansion of Escherichia-Shigella in IgAN (19, 43–47), but none of those studies particularly addressed the association of Escherichia-Shigella with GdIgA1 production or anti–Escherichia-Shigella antibodies in IgAN. As when we were preparing the manuscript, a new publication that followed a similar 16S rRNA approach had reached a similar conclusion on Escherichia-Shigella in association with IgAN (37). This study by Zhao et al. also addressed the prospective aspect of the gut microbiota in patients undergoing immunosuppressive therapy, showing changes in Escherichia-Shigella abundance in patients who entered posttreatment remission (37). Our study here additionally addressed the association of Escherichia-Shigella with GdIgA1, including the detection of IgA1-type anti–Escherichia-Shigella antibodies in IgAN. Furthermore, to control the confounding factors due to dietary habits and living environment, our study also enrolled paired healthy family members. We found that patients with IgAN had a higher overall level of anti-stx2 IgA1 than HCs, whereas there was still considerable overlap between groups. This might be because of the short circulating half-time of IgA1. Further studies are needed to explore the mechanism in detail.

Admittedly, our study has some limitations. Lacking animal experiment, we could not prove robust causal relationship of gut Escherichia-Shigella and IgAN. Furthermore, 16S rRNA signatures revealed genus level taxa of the gut microbiota, missing out some species-level classifications. Therefore, our analyses do not have the resolution to pinpoint the bacterial species that are truly responsible for inciting the mucosal GdIgA1 response or catabolizing IgA in preventing them from reentering circulation via retrograde transportation. For instance, although we took a broad approach to categorize the M64 class of IgA-P and have the individual IgA-Ps assigned to the corresponding bacterial species, it is clear to us that the evolution of M64 IgA-Ps did not strictly follow divergent selection. Instead, some bacteria may have either gained the gene by horizontal gene transfer from distantly related bacterial species or lost the protease gene in members of closely related species. Therefore, future animal studies should focus on more detailed classification of the gut microbiota at species, or even subspecies, level involvement in the IgAN pathogenesis. With regard to the IgA-Ps, classification should be based on primer sets that can distinguish M64 IgA-P sequences, as opposed to only broadly characterizing the genera based on 16S rRNA signatures.

In conclusion, our study, for the first time, demonstrated that Escherichia-Shigella exposure could possibly augment GdIgA1 production. Meanwhile, commensal bacteria of the Prevoltella genus and Clostridium genus that express M64-type of IgA proteases might participate in the natural catabolism of secreted IgA, leading to the overall reduction of GdIgA1 levels. Thus, our findings provide new perspective on the interplays among gut microbiome, mucosal infections, and mucosal immune responses during the onset and the progression of IgAN.

## Data availability statement

The datasets presented in this study can be found in online repositories. The names of the repository/repositories and accession number(s) can be found in the article/**Supplementary Material**.

## Ethics statement

The studies involving humans were approved by Medical ethics committee at the First Affiliated Hospital of Xi’an Jiaotong University. The studies were conducted in accordance with the local legislation and institutional requirements. Written informed consent for participation in this study was provided by the participants’ legal guardians/next of kin.

## Author contributions

LG: Data curation, Formal analysis, Methodology, Resources, Software, Validation, Visualization, Writing – original draft. HuL: Data curation, Formal analysis, Methodology, Resources, Software, Validation, Visualization, Writing – original draft. XL: Data curation, Methodology, Writing – original draft. PL: Data curation, Methodology, Writing – original draft. WL: Resources, Supervision, Writing – review & editing. HaL: Funding acquisition, Supervision, Validation, Writing – review & editing. JL: Supervision, Writing – review & editing. JJ: Project administration, Funding acquisition, Supervision, Writing – review & editing. XX: Conceptualization, Funding acquisition, Project administration, Supervision, Writing – review & editing.
