# Supplementary material for: Humoral immune responses primed by the alteration of gut microbiota were associated with galactose-deficient IgA1 production in IgA nephropathy
Source: Front Immunol. 2024 Jul 22;15:1415026. doi: 10.3389/fimmu.2024.1415026 (PMC11298704; doi:10.3389/fimmu.2024.1415026)
Supplement: Supplementary file 1 [file DataSheet_1.pdf]

Supplementary materials

Humoral immune responses primed by the alteration of gut microbiota were associated with galactose deficient IgA1 production in IgA nephropathy

Li Gao, Huixian Li, Xiaoling Liu, Haiyun Li, Wanhong Lu, Xinfang Xie, Jicheng Lv, Jing Jin

Table of Contents

Supplementary methods

Supplementary Tables

Table S1. Relative abundances of different genera in IgAN group, patients' house-matched healthy controls and non-related healthy controls.

Table S2. ROC analyses demonstrated Escherichia-Shigella had the best AUC value among other gut genera in differentiation cases between IgAN patients and healthy controls.

Table S3. Differentiation the macrofloral profiles among IgAN patients, patients' house-matched healthy controls and non-related healthy controls by logarithmic linear discriminant analysis (LDA) effect size (LefSe) threshold > 3.0.

Table S4. Differentiation the macrofloral profiles among paired IgAN patients and patients' house-matched healthy controls by logarithmic linear discriminant analysis (LDA) effect size (LefSe) with threshold > 3.0.

Table S5. Table S5. Genera of Prevotella and Alloprevotella were predicted with potential IgA proteases activity in UniProt database.

Supplementary Figures

Fig. S1. Compositions of gut microflora in IgA nephropathy and in healthy controls.

Fig. S2. Indicator species analysis including principal co-ordinate analysis (PCoA) analysis, heat map of genera abundances, ROC curve, and Indicator analysis were used to identify specific genus between IgAN patients and their paired house-matched healthy controls.

Fig. S3. Histogram of the linear discriminant analysis (LDA) scores (log10) for specific gut microbes with different abundances among involved groups.

Fig. S4. Characterization of gut microbiota differences between paired IgAN patients and their House-matched healthy controls by LEfSe analysis and LAD scores.

Fig. S5. Tax4fun functional analysis and KEGG pathway analysis of level 3 predicted higher proportions of pathogenic Eschrichia-coli infection in IgAN group than that in HC (A) and HM-HC (B) groups.

Fig. S6. Alignment of AK183_Peptidase_M64 sequences from Merops.

Fig. S7. Hypothesis of the causal role of the gut-kidney axis in IgA nephropathy.

Supplementary Methods

16S ribosomal RNA (rRNA) gene sequencing and data preprocessing.

Microbial DNA was extracted from the fecal samples using HiPure Soil DNA-extraction kit (Magen, Guangzhou, China) following manufacturer's standard protocol. The V3-V4 hypervariable region of the 16S rRNA genes was amplified by PCR using primer pair 341F (5'-CCTACGGGNGGCWGCAG -3') and 806R (5'-GGACTACHVGGGTATCTAAT-3'). Then the amplified 16S rRNA PCR fragments were sequenced using Illumina Nova SP (PE250) at Gene Denovo Biotechnology Co., Ltd (Guangzhou, China). To further improve read quality, raw reads were filtered based to a set of rules (operated using FASTP, version 0.18.0)[1]. The clean tags were clustered into operational taxonomic units (OTUs) of ≥97 % similarity using UPARSE[2] (version 9.2.64). Bioinformatic analyses, including alpha diversity analysis, beta diversity analysis, community composition analysis, indicator species analysis, and function prediction were all performed using Omicsmart, a real-time interactive online platform for data analysis (http:// www.omicsmart.com). Additional methods on 16S rRNA gene sequencing are in supplementary materials. DateBase:http://www.ncbi.nlm.nih.gov/bioproject/1125592 (BioProject ID: PRJNA1125592)

Bioinformatics analysis for 16S rRNA sequencing data Alpha diversity analysis

Chao1, ACE, Shannon, Simpson, Good's coverage were calculated in QIIME[3] (version 1.9.1). OTU rarefaction curve and rank abundance curves were plotted in R project ggplot2 package (version 2.2.1). Alpha index comparison between groups was calculated by Welch's t-test and Wilcoxon rank test in R project Vegan package[4](version 2.5.3). Alpha index comparison among groups was computed by Tukey's HSD test and Kruskal-Wallis H test in R project Vegan package[4](version 2.5.3).

Beta diversity analysis

Sequence alignment was performed using Muscle[5] (version 3.8.31) and phylogenetic tree was constructed using FastTree[6](version 2.1). PCA (principal component analysis) was performed in R project Vegan package [4](version 2.5.3).Multivariate statistical techniques including PCoA (principal coordinates analysis) of Unweighted unifrac distances were generated in R project Vegan package[4] (version 2.5.3)and plotted in R project ggplot2 package [7](version 2.2.1). Statistical analysis of Welch's t-test, Wilcoxon rank test, and Kruskal-Wallis H test were calculated in R project Vegan package[4](version 2.5.3).

Community composition analysis

The abundance statistics of each taxonomy was visualized using Krona[8](version 2.6).The stacked bar plot of the community composition was visualized in R project ggplot2 package[7] (version 2.2.1). Heatmap of species abundance was plotted using pheat-map package (version 1.0.12) in R project [9].

Indicator species analysis

Between groups Venn analysis was performed in R project VennDiagram package (version 1.6.16)[10] Species comparison among groups was computed by tukey's HSD test and kruskal-wallis H test in R project Vegan package[7](version 2.5.3). Biomarker features in each group were screened by LEfSe software[11](version 1.0), pROC package[12] (version 1.10.0) in R project. Indicator values were calculated based on the abundance and frequency of genus by labdsv package (version2.0-1) in R project and differences of Indicator values between groups were test by cross-validation.

Function prediction

The KEGG pathway analysis of the OTUs was inferred using Tax4Fun[13](version 1.0). Microbiome phenotypes of bacteria were classified using BugBase. Analysis of function difference between groups was calculated by Welch's t-test, Wilcoxon rank test and Kruskal-Wallis H test in R project Vegan package (version 2.5.3).

Table S1. Relative abundances of different genera in IgAN group, patients' house-matched healthy controls and non-related healthy controls.

	Relative abundance (%)		
Genus	HM-HC	HC	IgAN
Bacteroides	22.13	19.14	27.85
Faecalibacterium	12.45	13.50	11.42
Megamonas	14.28	7.74	7.79
Prevotella_9	13.11	8.79	2.06
Escherichia-Shigella	1.32	2.36	8.07
Phascolarctobacterium	1.46	3.40	2.21
Parabacteroides	0.54	1.58	2.39
Lachnoclostridium	2.00	2.43	1.43
Dialister	2.38	1.82	1.70
Bifidobacterium	0.60	1.70	1.76
Other	22.81	28.62	26.92
Unclassified	6.91	8.91	6.38

HM-HC, household-matched healthy control; HC, healthy control; IgAN, IgA nephropathy.

Table S2. ROC analyses demonstrated Escherichia-Shigella had the best AUC value among other gut genera in differentiation cases between IgAN patients and healthy controls.

Factor	AUC	95% CI (AUC)	best_thresholds (specificities,sensitivities)
Bacteroides	0.649	0.55~0.748	24.6952 (0.787,0.468)
Prevotella_9	0.696	0.604~0.787	0.22615 (0.83,0.545)
**Escherichia-Shigella**	**0.837**	**0.76~0.915**	**0.68955 (0.681,0.896)**
Parabacteroides	0.664	0.565~0.763	1.12515 (0.702,0.584)
Subdoligranulum	0.642	0.542~0.743	0.7098 (0.702,0.519)
Megasphaera	0.786	0.707~0.865	0.0435 (0.83,0.714)
Lachnospira	0.659	0.561~0.757	0.65535 (0.532,0.753)
Alistipes	0.613	0.51~0.715	0.0389 (0.255,0.987)
Ruminococcus_torques_group	0.701	0.603~0.798	0.17245 (0.596,0.766)
Ruminococcaceae_UCG-005	0.619	0.511~0.726	0.02195 (0.404,0.909)
Ruminococcus_gnavus_group	0.725	0.63~0.82	0.02275 (0.404,0.961)
Alloprevotella	0.801	0.722~0.879	0.0016 (0.894,0.714)
Butyricicoccus	0.689	0.59~0.789	0.2364 (0.66,0.701)
Prevotellaceae_UCG-001	0.742	0.651~0.833	0.0078 (0.766,0.714)
Sutterella	0.676	0.578~0.773	0.01115 (0.872,0.416)
Lachnospiraceae_UCG-004	0.691	0.593~0.79	0.16795 (0.702,0.662)
Flavonifractor	0.662	0.558~0.765	0.08775 (0.638,0.675)
Hungatella	0.793	0.709~0.878	0.00895 (0.553,0.922)

IgAN, IgA nephropathy.

Table S3. Differentiation the macrofloral profiles among IgAN patients, patients' house-matched healthy controls and non-related healthy controls by logarithmic linear discriminant analysis (LDA) effect size (LefSe) threshold > 3.0.

Feature	Level	Log_hm	Class_hm	LDA score	p value
Prevotellaceae	Family	5.198027	HM-HC	4.839979	6.52E-06
Prevotella_9	Genus	5.099045	HM-HC	4.741801	4.38E-07
Enterobacteriales	Order	4.986702	IgAN	4.58378	9.84E-07
Enterobacteriaceae	Family	4.986702	IgAN	4.58378	9.84E-07
Escherichia_Shigella	Genus	4.930491	IgAN	4.569327	1.91E-09
Proteobacteria	Phylum	5.074938	IgAN	4.538013	0.000605
Megamonas	Genus	5.135095	HM-HC	4.530566	0.030806
Gammaproteobacteria	Class	5.051866	IgAN	4.52066	0.00138
Bacteroides_ovatus_CL02 T12C04	Species	4.486559	IgAN	4.044488	0.001132
Alloprevotella	Genus	4.305084	HM-HC	4.036982	1.17E-16
Tannerellaceae	Family	4.383572	IgAN	3.970487	5.26E-05
Parabacteroides	Genus	4.38349	IgAN	3.970386	5.36E-05
Bacteroides_fragilis	Species	4.261323	IgAN	3.866463	8.06E-06
Bacteroides_thetaiotaomi cron	Species	4.196461	IgAN	3.817246	1.65E-06
Muribaculaceae	Family	4.105481	HM-HC	3.750431	3.92E-05
Megasphaera	Genus	4.207901	HC	3.7488	1.29E-06
Lachnospira	Genus	4.237665	HC	3.684149	0.000308
Bacteroides_coprocola_DS M_17136	Species	4.295227	HM-HC	3.683395	0.039896
Prevotellaceae_UCG_001	Genus	3.987117	HM-HC	3.646332	4.80E-14
Prevotellaceae_NK3B31_g roup	Genus	3.849152	HC	3.600995	9.56E-23
Prevotella_sp_109	Species	3.848726	HC	3.600892	1.54E-24

Ruminococcus_torques_group	Genus	3.906178	IgAN	3.536923	2.04E-07
Ruminococcus_gnavus_group	Genus	3.904229	IgAN	3.529228	8.79E-06
Oceanospirillales	Order	3.680383	HM-HC	3.429955	2.82E-11
Veillonella_ratti	Species	3.805661	IgAN	3.421324	1.49E-06
Halomonadaceae	Family	3.661325	HM-HC	3.4143	1.98E-13
Halomonas	Genus	3.652343	HM-HC	3.405917	2.43E-15
Bifidobacterium_longum_subsp_longum	Species	3.790765	IgAN	3.398418	0.000772
Mitsuokella	Genus	3.689453	HM-HC	3.36412	1.69E-20
Parabacteroides_distasonis	Species	3.756193	IgAN	3.327358	0.001765
Hungatella	Genus	3.64066	IgAN	3.297585	6.90E-11
Butyricicoccus	Genus	3.680691	HC	3.282494	0.002264
Streptococcaceae	Family	3.676377	HC	3.241204	0.030709
Streptococcus	Genus	3.67212	HC	3.238217	0.027803
Leuconostocaceae	Family	3.616062	HC	3.22386	1.35E-06
Streptococcus_salivarius_subsp_thermophilus	Species	3.616023	HC	3.201981	0.037956
Lachnoclostridium_sp_YL32	Species	3.66794	IgAN	3.17891	0.000607
Leuconostoc	Genus	3.549931	HC	3.163893	1.12E-11
Leuconostoc_lactis	Species	3.534132	HC	3.141853	2.31E-14
Ruminococcaceae.UBA1819	Genus	3.442001	IgAN	3.088454	2.47E-08
Bacteria.Proteobacteria.Deltaproteobacteria	Class	3.640606	IgAN	3.086246	0.024459
Flavonifractor	Genus	3.500562	IgAN	3.070421	0.000193
Desulfovibrionales	Order	3.606661	IgAN	3.066044	0.036706
Desulfovibrionaceae	Family	3.606661	IgAN	3.066044	0.036706
Methylomonaceae	Family	3.416552	HC	3.036091	0.012607

Methyloprofundus	Genus	3.416552	HC	3.036091	0.011813
Tyzzerella_4	Genus	3.343876	IgAN	3.015365	3.98E-05

HM-HC, household-matched healthy control; HC, healthy control; IgAN, IgA nephropathy.

Table S4. Differentiation the macrofloral profiles among paired IgAN patients and patients' house-matched healthy controls by logarithmic linear discriminant analysis (LDA) effect size (LefSe) with threshold > 3.0.

Feature	Level	Log_hm	Class_hm	LDA score	p value
Prevotellaceae	Family	5.216676	HM-HC	4.818883	0.001892
Prevotella_9	Genus	5.117662	HM-HC	4.723006	0.000153
Enterobacteriales	Order	5.058956	IgAN	4.654031	0.000859
Enterobacteriaceae	Family	5.058956	IgAN	4.654031	0.000859
Proteobacteria	Phylum	5.14054	IgAN	4.62323	0.013218
Escherichia_Shigella	Genus	4.981516	IgAN	4.605861	0.000169
Gammaproteobacteria	Class	5.118091	IgAN	4.602753	0.009924
Megamonas	Genus	5.154925	HM-HC	4.387797	0.033532
Alloprevotella	Genus	4.32385	HM-HC	4.057659	1.40E-06
Bacteroides_fragilis	Species	4.437841	IgAN	3.957656	9.16E-05
Bacteroides_ovatus_CL02T12C04	Species	4.342798	IgAN	3.825994	0.029557
Prevotellaceae_UCG_001	Genus	4.006659	HM-HC	3.74347	3.16E-05
Lachnospiraceae.Lachnospira	Genus	4.236158	HM-HC	3.641707	0.017444
Burkholderiaceae	Family	4.226443	HM-HC	3.568995	0.031491
Ruminococcus_torques_group	Genus	3.892385	IgAN	3.544446	5.61E-06
Megasphaera	Genus	4.104464	HM-HC	3.492073	0.015202
Tannerellaceae	Family	4.044189	IgAN	3.472356	0.016289
Parabacteroides	Genus	4.044144	IgAN	3.472265	0.016289
Lachnoclostridium_sp_YL32	Species	3.879853	IgAN	3.447546	0.016289
Parasutterella	Genus	4.051925	HM-HC	3.429812	0.024358
Oceanospirillales	Order	3.695239	HM-HC	3.418925	7.39E-05
Halomonadaceae	Family	3.679492	HM-HC	3.410196	1.54E-06
Halomonas	Genus	3.670467	HM-HC	3.401255	1.39E-06
Mitsuokella	Genus	3.709614	HM-HC	3.398689	4.30E-09
Ruminococcus_gnavus_group	Genus	3.733839	IgAN	3.344852	0.002874
Bacteroides_thetaiotaomicron	Species	3.974443	HM-HC	3.32381	0.010671
Bifidobacterium_longum_subsp_longum	Species	3.705878	IgAN	3.30393	0.002059
Legionellales	Order	1.308616	IgAN	3.246432	0.038061
Massiliprevotella_massiliensis	Species	3.422203	IgAN	3.240074	0.018885
Prevotella_2	Genus	3.460141	IgAN	3.205777	0.000142
Hungatella	Genus	3.379394	IgAN	3.083559	1.60E-05
Flavonifractor	Genus	3.559164	IgAN	3.047486	0.009924

Comamonas	Genus	3.351391	HM-HC	3.045851	8.33E-05

HM-HC, household-matched healthy control; HC, healthy control; IgAN, IgA nephropathy.

Table S5. Genera of Prevotella and Alloprevotella were predicted with potential IgA proteases activity in UniProt database.

Entry	Entry Name	Gene Names	Organism
A0A069QNU1	A0A069QNU1_PRELO	HMPREF1991_00256	Prevotella loescheii DSM 19665
A0A095ZID5	A0A095ZID5_9BACT	HMPREF2137_09320	Prevotella buccalis DNF00853
A0A0D9NBJ2	A0A0D9NBJ2_PREIN	M573_125018	Prevotella intermedia ZT
A0A0K1NI79	A0A0K1NI79_9BACT	ADJ77_02570	Prevotella fusca JCM 17724
A0A0S2KM93	A0A0S2KM93_9BACT	AS203_08675	Prevotella enoeca
A0A0T7ANT8	A0A0T7ANT8_PREIN	PIOMA14_II_0193	Prevotella intermedia
A0A133PT44	A0A133PT44_9BACT	HMPREF3226_02871	Prevotella corporis
A0A134BXF5	A0A134BXF5_9BACT	HMPREF3034_00777	Prevotella sp. DNF00663
A0A137SZD7	A0A137SZD7_9BACT	HMPREF3202_00589	Prevotella bivia
A0A1B1I854	A0A1B1I854_9BACT	AXF22_05760	Prevotella scopos JCM 17725
A0A1I2QR96	A0A1I2QR96_9BACT	SAMN05216383_10982	Prevotella sp. KH2C16
A0A1P8JNM7	A0A1P8JNM7_PREIN	BWX40_10730	Prevotella intermedia
A0A2A6EH00	A0A2A6EH00_PREIN	CLI71_02655	Prevotella intermedia
A0A2D3L8Y3	A0A2D3L8Y3_PREIN	CTM62_09310	Prevotella intermedia
A0A2D3LMH0	A0A2D3LMH0_PREIN	CTM46_09650	Prevotella intermedia
A0A2D3MGD0	A0A2D3MGD0_PREIN	CTM44_11775	Prevotella intermedia
A0A2D3N912	A0A2D3N912_PREIN	CTM50_01825	Prevotella intermedia
A0A2G8I1F6	A0A2G8I1F6_PREIN	CUB95_10055	Prevotella intermedia
A0A2K9HA68	A0A2K9HA68_9BACT	CRM71_04245	Prevotella jejuni
A0A2N6Q6L4	A0A2N6Q6L4_9BACT	CJ232_04650	Prevotella timonensis
A0A379E2Z7	A0A379E2Z7_9BACT	NCTC13067_00728	Prevotella denticola
A0A379GBG1	A0A379GBG1_9BACT	NCTC13043_02351	Prevotella pallens
A0A3E4SCN7	A0A3E4SCN7_9BACT	DXC61_11810	Prevotella copri
A0A3R6G193	A0A3R6G193_9BACT	DW060_10675	Prevotella stercorea
A0A3R6JTD6	A0A3R6JTD6_9BACT	DW026_02260	Prevotella copri
A0A3S4TFB6	A0A3S4TFB6_9BACT	NCTC13071_01676	Prevotella oris
A0A412VBG9	A0A412VBG9_9BACT	DWW35_00425	Prevotella copri
A0A432LII4	A0A432LII4_9BACT	EHV08_03800	Prevotella koreensis
A0A5P0VIW4	A0A5P0VIW4_9BACT	F7D97_04800	Prevotella copri
A0A646G760	A0A646G760_9BACT	F7D83_11785	Prevotella copri
A0A660NXP5	A0A660NXP5_9BACT	D8B57_03895	Prevotella sp
A0A7D4KDI2	A0A7D4KDI2_9BACT	FIU21_07205	Prevotella melaninogenica
A0A7H1MRY3	A0A7H1MRY3_9BACT	FO447_06525	Prevotella copri
A0A7H1MWL4	A0A7H1MWL4_9BACT	FO447_08010	Prevotella copri
A0A8G0HDG1	A0A8G0HDG1_9BACT	GRF55_05305	Prevotella sp. Rep29
C9LIE8	C9LIE8_9BACT	GCWU000325_02007	Alloprevotella tannerae ATCC 51259

C9MPI1	C9MPI1_9BACT	HMPREF0973_01522	Prevotella veroralis F0319
C9PZ55	C9PZ55_9BACT	HMPREF6745_2228	Prevotella sp. oral taxon 472 str. F0295
D1PI05	D1PI05_9BACT	F7D21_01875<br>PREVCOP_06882	Prevotella copri DSM 18205
D1VWI6	D1VWI6_9BACT	HMPREF9019_0833	Prevotella timonensis CRIS 5C-B1
D1W524	D1W524_9BACT	HMPREF0650_0801	Prevotella buccalis ATCC 35310
D3HW89	D3HW89_9BACT	HMPREF0649_00521	Prevotella buccae D17
D3IHH5	D3IHH5_9BACT	HMPREF0670_00794	Prevotella sp. oral taxon 317 str. F0108
D7N9K2	D7N9K2_9BACT	HMPREF0665_00331	Prevotella oris C735
D9RRU5	D9RRU5_PREMB	HMPREF0659_A5623	Prevotella melaninogenica (strain ATCC 25845 / DSM 7089 / JCM 6325 / VPI 2381 / B282) (Bacteroides melaninogenicus)
E1KT16	E1KT16_9BACT	HMPREF9296_0491	Prevotella disiens FB035-09AN
E6K7L1	E6K7L1_9BACT	HMPREF6485_1857	Prevotella buccae ATCC 33574
E6ML44	E6ML44_9BACT	HMPREF9420_0211	Prevotella salivae DSM 15606
E7RT91	E7RT91_9BACT	HMPREF0663_11964	Prevotella oralis ATCC 33269
F0H521	F0H521_9BACT	HMPREF9303_2507	Prevotella denticola CRIS 18C-A
F9DE61	F9DE61_9BACT	HMPREF9419_2374	Prevotella nigrescens ATCC 33563
F9DKU8	F9DKU8_9BACT	HMPREF9144_2290	Prevotella pallens ATCC 700821
G6AYI1	G6AYI1_9BACT	HMPREF0673_01691	Prevotella stercorea DSM 18206
I4Z6I2	I4Z6I2_9BACT	PrebiDRAFT_0020	Prevotella bivia DSM 20514
L1NH34	L1NH34_9BACT	HMPREF9151_00744	Prevotella saccharolytica F0055
R5CK14	R5CK14_9BACT	BN567_00203	Prevotella sp. CAG:255
R5CSI4	R5CSI4_9BACT	BN458_00215	Prevotella sp. CAG:1058
R5FLL1	R5FLL1_9BACT	BN812_01296	Prevotella sp. CAG:924
R5ZTE8	R5ZTE8_9BACT	BN693_00600	Prevotella sp. CAG:5226
R6BBA2	R6BBA2_9BACT	BN731_00618	Prevotella sp. CAG:604
R6BP68	R6BP68_9BACT	BN510_01373	Prevotella copri CAG:164
R6E5Z1	R6E5Z1_9BACT	BN487_01379	Prevotella sp. CAG:1320
R6EWQ3	R6EWQ3_9BACT	BN691_01934	Prevotella sp. CAG:520
R6PSK0	R6PSK0_9BACT	BN637_00381	Prevotella sp. CAG:386
R6VXZ9	R6VXZ9_9BACT	BN725_00899	Prevotella sp. CAG:592
R6XXE3	R6XXE3_9BACT	BN769_01014	Prevotella sp. CAG:732
R7H774	R7H774_9BACT	BN741_00314	Prevotella stercorea CAG:629
R7LDP2	R7LDP2_9BACT	BN805_00883	Prevotella sp. CAG:891
R7P8S2	R7P8S2_9BACT	BN736_00012	Prevotella sp. CAG:617
U2J5U6	U2J5U6_9BACT	HMPREF9148_01998	Prevotella sp. F0091
U2KQG0	U2KQG0_9BACT	HMPREF9145_2068	Prevotella salivae F0493
U2MPV3	U2MPV3_9BACT	HMPREF1218_0019	Prevotella pleuritidis F0068
U2NMB1	U2NMB1_9BACT	HMPREF9135_1925	Prevotella baroniae F0067
U7UGP7	U7UGP7_9BACT	HMPREF1254_0791	Prevotella sp. BV3P1
X6PV04	X6PV04_9BACT	HMPREF1505_1552	Prevotella sp. ICM33
R5NHP1	R5NHP1_9BACT	BN471_00279	Paraprevotella clara CAG:116

A0A496KGJ1	A0A496KGJ1_9BACT	D8H91_09950	Alloprevotella sp
L1MAY5	L1MAY5_9BACT	HMPREF9999_01902	Alloprevotella sp. oral taxon 473 str. F0040
C9LIE8	C9LIE8_9BACT	GCWU000325_02007	Alloprevotella tannerae ATCC 51259

Species from fecal collections were shown as the highlight.

References

1. Wu H, Irizarry RA, Bravo HC: Intensity normalization improves color calling in SOLiD sequencing. *Nat Methods,* 7**:** 336-337, 2010

2. Oksanen J BFG, Kindt R, et al: Vegan: community ecology package. R package version 1.17 4[J]. http://cran. r project. org>. *Acesso em, ,* 23: 2010.

3. Edgar RC: MUSCLE: multiple sequence alignment with high accuracy and high throughput. *Nucleic Acids Res,* 32**:** 1792-1797, 2004

4. Price MN, Dehal PS, Arkin AP: FastTree 2--approximately maximum-likelihood trees for large alignments. *PLoS One,* 5**:** e9490, 2010

5. Wickham HgWIRCS: Wickham, H. (2011). ggplot2. Wiley Interdisciplinary Reviews: Computational Statistics, 3(2),180 185. doi:10.1002/wics.147.

6. Ondov BD, Bergman NH, Phillippy AM: Interactive metagenomic visualization in a Web browser. *BMC Bioinformatics,* 12**:** 385, 2011

7. Kolde R, Kolde M R. Package ‘pheatmap’[J]. R Package, 2015, 1(7).

8. Chen H, Boutros PC: VennDiagram: a package for the generation of highly-customizable Venn and Euler diagrams in R. *BMC Bioinformatics,* 12**:** 35, 2011

9. Segata N, Izard J, Waldron L, Gevers D, Miropolsky L, Garrett WS, et al.: Metagenomic biomarker discovery and explanation. *Genome Biol,* 12**:** R60, 2011

10. Robin X, Turck N, Hainard A, Tiberti N, Lisacek F, Sanchez JC, et al.: pROC: an open-source package for R and S+ to analyze and compare ROC curves. *BMC Bioinformatics,* 12**:** 77, 2011

11. Asshauer KP, Wemheuer B, Daniel R, Meinicke P: Tax4Fun: predicting functional profiles from metagenomic 16S rRNA data. *Bioinformatics,* 31**:** 2882-2884, 2015

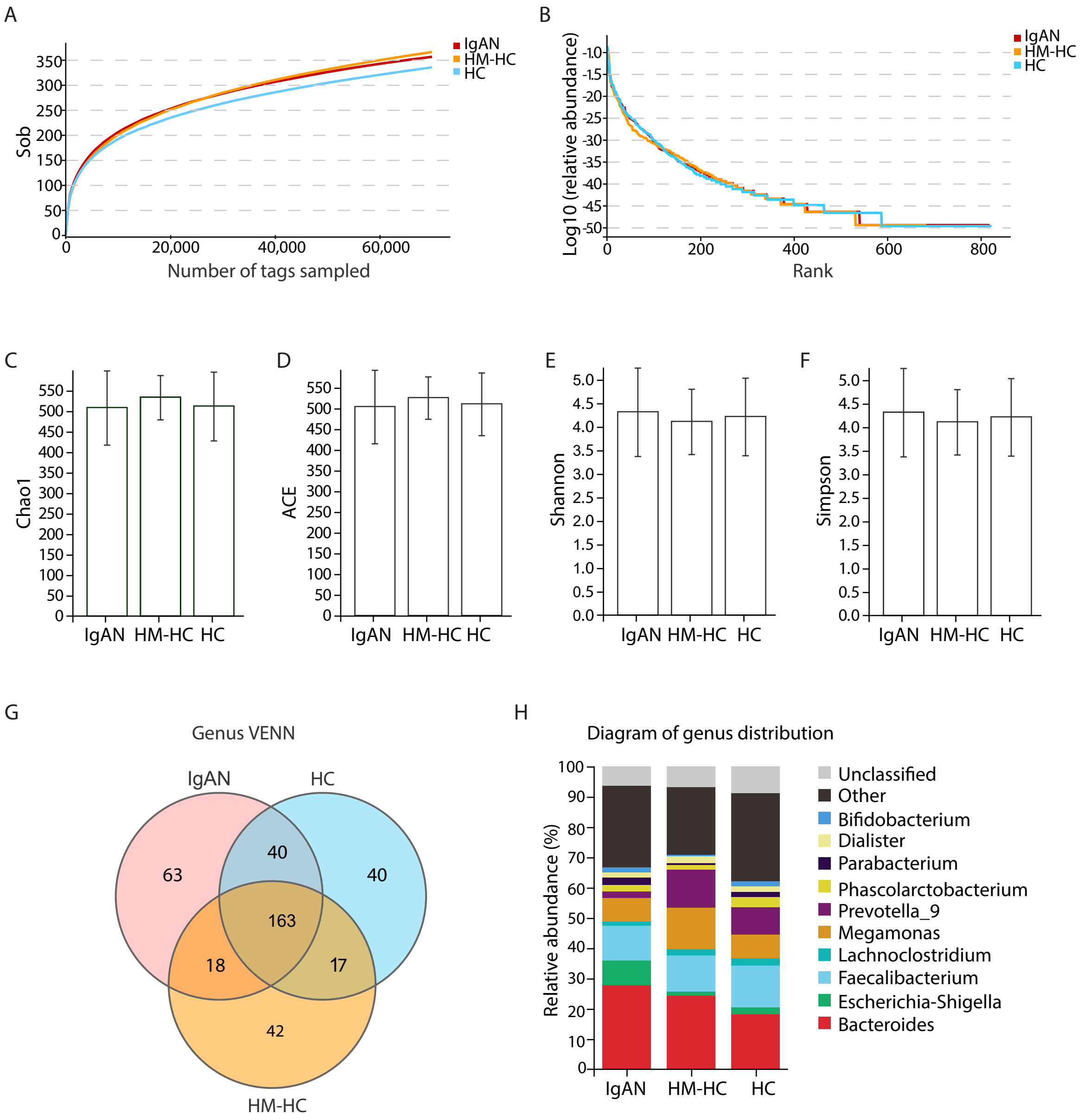

Fig. S1. Compositions of gut microflora in IgA nephropathy and in healthy controls.
The rarefaction curves indicated sufficient depth and a reasonable sequencing volume (A) among the IgAN, the HM-HC, and the HC groups. Rank abundance curves showed taxa richness and evenness among the groups (B). There was no significant difference in alpha diversity analyses of Chao1, ACE, Shannon, Simpson between IgAN and the controls, as calculated by Welch's t-test and Wilcoxon rank test (C-F). The Venn diagram showed the overlaps of bacterial genera among the IgAN and the two control groups(G). The profiles of fecal microbiota genera showed 12 most abundant genera in patients with IgAN, as compared to those of the HM-HC and the HC groups. HM-HC, household-matched healthy control; HC, healthy control; IgAN, IgA nephropathy.

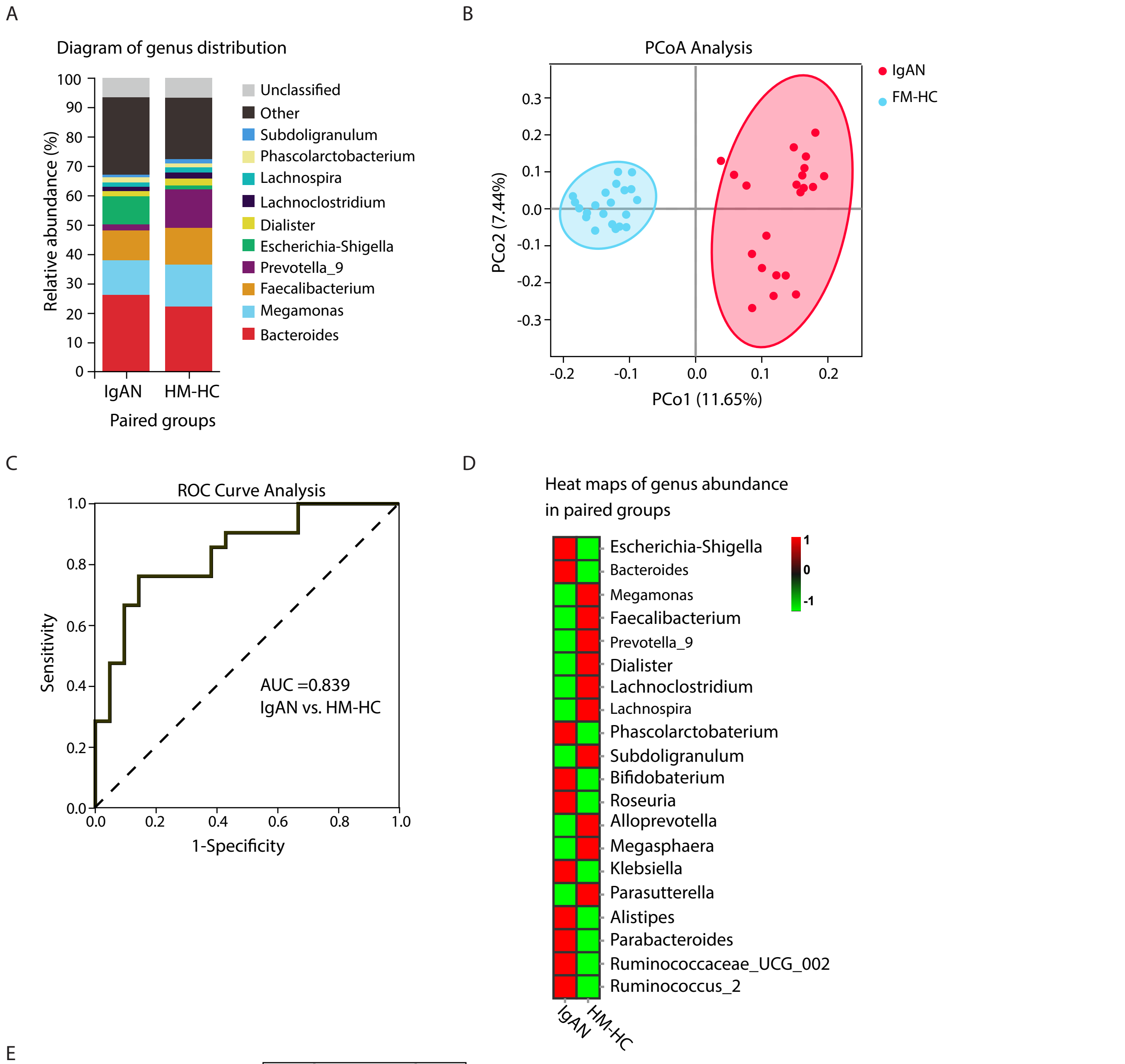

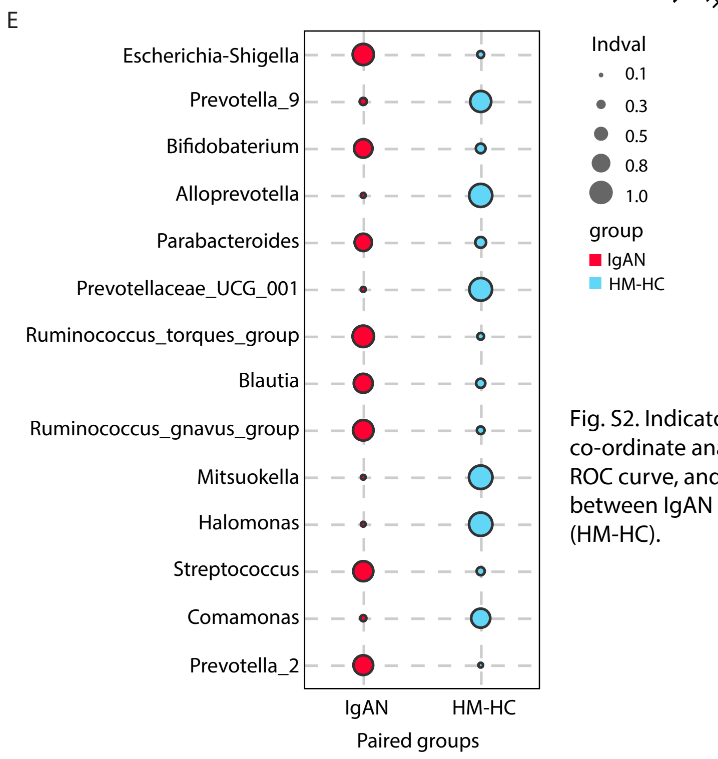

Fig. S2. Indicator species analysis including principal co-ordinate analysis (PCoA) analysis, heat map of genera abundances, ROC curve, and Indicator analysis were used to identify specific genus between IgAN patients and thier paired house-matched healthy controls (HM-HC).

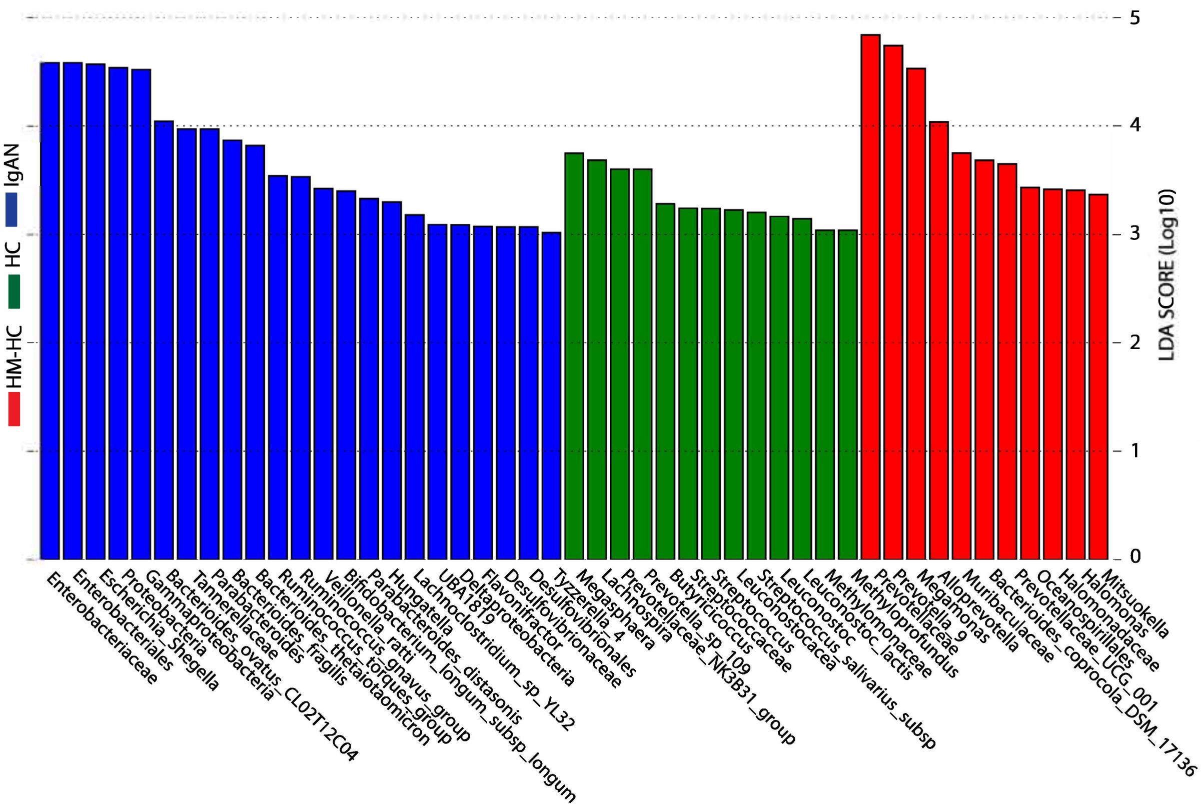

Fig. S3. Histogram of the linear discriminant analysis (LDA) scores (log10) for specific gut microbes with different abundances among involved groups.

23 enriched taxa were identified as specific markers in patients with IgAN (bule) with threshold > 3.0 , while 13 taxa (green) enriched in HC group and 11 taxa (red) enriched in HM-HC group were identified with significant differences from those in IgAN. See LDA values of features in Table S3.

HM-HC, household-matched healthy control; HC, healthy control; IgAN, IgA nephropathy.

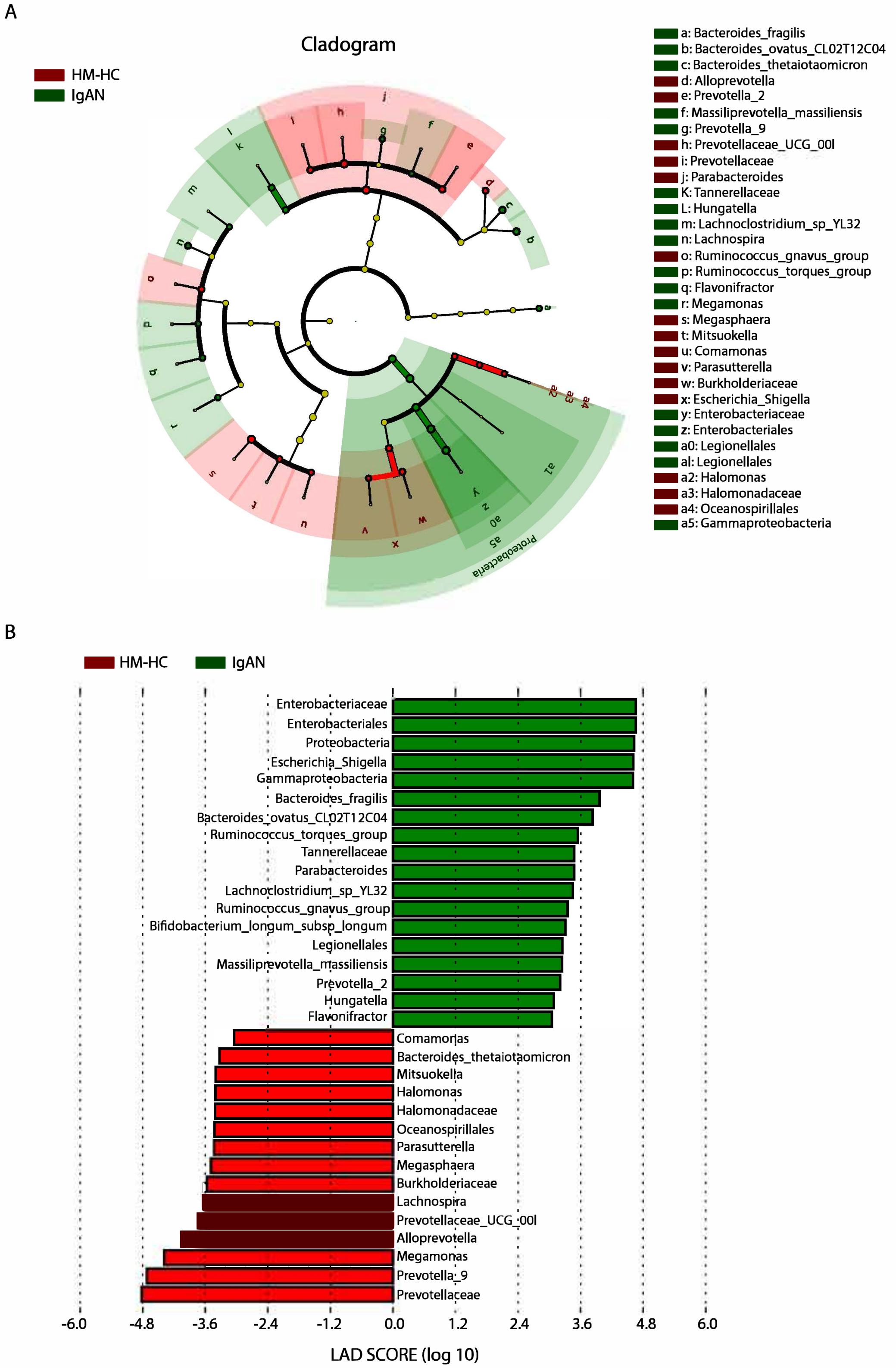

Fig. S4. Characterization of gut microbiota differences between paired IgAN patients and thier House-matched healthy controls by LEfSe analysis and LAD scores.
A. Cladograms of bacterial lineages at various levels from phyla to species with significant differences between IgAN and HM-HC were shown. B. 18 significantly enriched bacterial taxa were identified in patients with IgAN (green) with threshold > 3.0. Among them Escherichia-Shigella with higher LDA values was ont of the best markers. See LDA values of features in Table S4.

LEfSe, linear discriminant analysis linear discriminant analysis (LDA) effect size; HM-HC, and HC. HM-HC, household-matched healthy control; HC, healthy control; IgAN, IgA nephropathy.

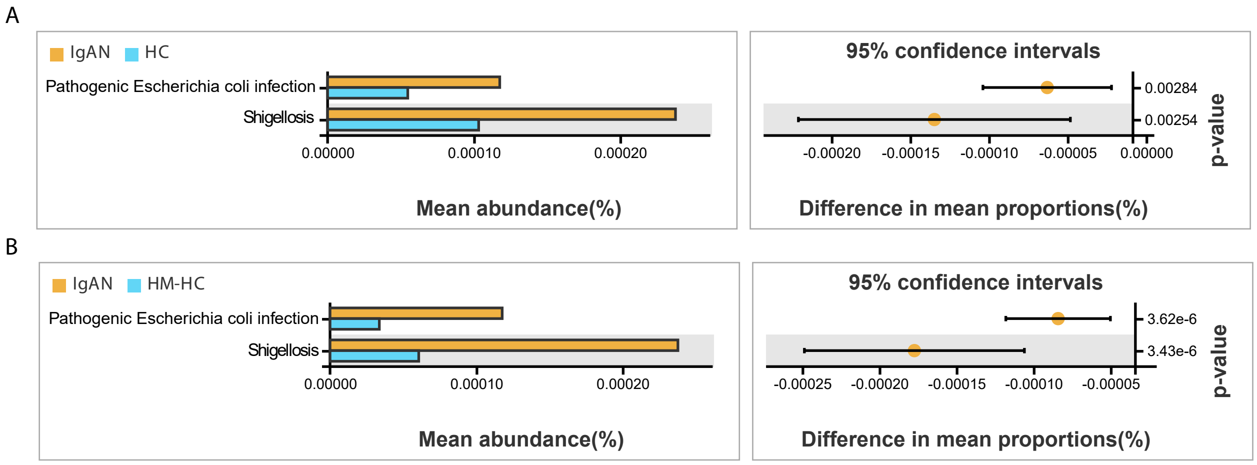

Fig. S5. Tax4fun functional analysis and KEGG pathway analysis of level 3 predicted higher proportions of pathogenic Eschrichia-coli infection in IgAN group than that in HC (A) and HM-HC (B) groups.

HM-HC, household-matched healthy control; HC, healthy control; IgAN, IgA nephropathy.

MER067561	-------PKAKLLRGRHDGKGVDLVIAGEAYTASMQ---DELLEDAQLVDKYAFDDFEEI	50
MER168445	-YN--TGDVIVYQ-RMRTENPVTMVVTGDGYTADDLLEGGLFERRAREALDFLF-TVEPY	55
MER310622	----PRCETFEVMYSGNSAQRVDIVLLPEGYGAGER---AKFESACRTFADEFF-SYSPY	52
MER384775	----PRCETFEVMYSGNSAQRVDIVLLPEGYGAGER---AKFESACRTFADEFF-SYSPY	52
MER384771	-------------------ECLDVLILGDGYSIKEE---EKFRKDLNHYTELMM-NFPPY	37
MER064266	-------PVIALQDSGDPTAKLDLLLVGDGYTAKDR---PKFEQDARRLLGTLF-EHEPF	49
MER066009	-------PLIEILKSGDPAHKLDLLLLGDGYTEAER---GKFEKDARRLVAELF-EHEPF	49
MER118905	-------PLIEILKSGDPAHKLDLLLLGDGYTEAER---GKFEKDARRLVAELF-EHEPF	49
MER162996	-----PAQPIPIRVSGPSPDKVDLLVLGDGYTQAEM---PKFEAAVRRLAQHLF-EVSPF	51
MER065708	SRPASPGALIELQKSGPSSDKVDLLVMGDGYTADER---GKFETDAKKFIETLF-ATSPF	56
MER141845	SSPPSPGALLPLLENGPPSEKVDLLILGDGYTAQER---AKFEKDARRMVDILF-TFSPF	56
MER068550	-------PLLKLLENGPSAQKVDLLILGDGYTKAEH---AKFEKDARHMVDILF-TFSPF	49
MER251522	-------PLLKLMENGPSAQKVDLLILGDGYTKAEH---AKFEKDARHMVDILF-TYAPF	49
MER191119	-YANVTVPVYTIQKPRSDKENMVIVYCAEGYTKSQQ---KQFVEDVKKLWGEVL-QIEPY	55
MER310605	-YANVTVPVYTIQKPRSDKENMVIVYCAEGYTKSQQ---KQFVEDVKKLWGEVL-QIEPY	55
MER310604	------IPVYTIQKTRSDTENMVVVVCGEGYTKSQQ---GKFINDVKRLWQDAM-KYEPY	50
MER191125	-YSNPTIPVYTIQKTRSDTENMVVVVCGEGYTKSQQ---GKFINDVKRLWQDAM-KYEPY	55
MER016067	-YSNPTIPVYTIQKTRSDTENMVVVVCGEGYTKSQQ---GKFINDVKRLWQDAM-KYEPY	55
MER105722	-YSNPTIPVYTIQKTRSDTENMVVVVCGEGYTKSQQ---GKFINDVKRLWQDAM-KYEPY	55
MER105702	-YSNPTIPVYTIQKTRSDTENMVVVVCGEGYTKSQQ---GKFINDVKRLWQDAM-KYEPY	55
MER191126	--SKTTFPVQVIHKTGDDKENFVIVIMGDGYTAGQQ---DQFLEDATQKARGML-TWSPY	54
MER013883	--------VVPIQVTGAPSERFNLVILGDGYTEEEM---PLFREQLDQHLNVQW-SIEPF	48
MER062296	--------VVPLQVTGPATSRFNLVVMGDGYTAAEL---PKFREQVDKHLNVLW-SIEPF	48
MER220607	--AEPAATVTEVQVTGPVAQRFNLVVLGDGYTAAEQ---PKFFADVERHVSTLW-SLEPF	54
MER193549	----------PVEVNGPSADRIDLVFVGDGYTEAEL---GLYADQVAAKWALLA-NREPF	46
MER019878	--------VVPLAETGPTADRLDVTIVGDGYTAAEQ---DDFLEDARAKWDEVT-DIDPY	48
MER310615	--------VVPLAETGPTADRLDVTIVGDGYTAAEQ---DDFLEDARAKWDEVT-DIDPY	48
MER289439	----------------STADRLDIVVVGDGYTEAEL---GAFRADAREKWAELT-AVEPY	40
MER179396	----------------STADRLDVVVIGDGYTAAEL---DRFHADARQKWAEVA-AVEPY	40
MER310638	-----------------------VVVIGDGYTAGEL---DRFHADARAKWAEVT-AVEPY	33
MER384778	-----------------------VVFIGDGYTAAQQ---EDFHADVRAKWAKMT-AVEPY	33
MER310639	--------VSAVIQTGPVADKLDVVFIGDGYTSAQQ---GDFHADLRSKWEQMS-AVEPY	48
MER310648	--------VASVIDNGTTADKLDVVFIGDGYTAGQQ---ADFQADARSKWDQMS-AVEPY	48
MER098980	--------VVTIQYSGLSSQRFDMVFIGDGYTATEM---DTLRKHAQAKWDEIA-ASAPW	48
MER131394	-------------------EKLDIVVVGDGYTEADM---ATYEEHTRSKMDEVF-AVEPF	37
MER310627	--------VVPIQQTGPSSQRFDLVFVGDGYTSAEL---GKYKQQATSRWNELT-QVEPF	48
MER163974	-------------KSLADEDAFVLLIFGDGFTASEQ---DSFYTNAQNTADYLM-DTSPW	43
MER310631	-----------------DKDALLLTFFGDGFTEKEQ---ELFFAEAKRMAKYMM-ATSPW	39
MER058697	--------VYSVFKTGDSENRADLVLLSEGYTNGDL---SKFEEDVRKIVEGYF-GEDIY	48
MER166461	----------------SPSEKIDLVLLGDGYTAGEM---DKFRHDAMQITDGFF-SKSPY	40
MER133451	--------VDTLYKNGPLDNRINVVILGDGFTEEQL---PKFAEEAKKFADFFL-AYNPY	48
MER059337	--	0
MER191123	--------IDTLQYQGTNKHIVNMVILADGYTAAEL---DYFVEDARRFNNYFF-NTEPF	48
MER173069	--	0
MER177104	--	0
MER203593	-------PHVTLQQAADTARCIRVAFVAEGYQQQEM---DVFLNDCRIAMESLF-EHEPF	49
MER310647	-------PYIFLQKATDTTRCIHIAFVAEGYTRAEM---PTFLADARRAMGYIF-SYEPF	49
MER310616	--------YELLQQAADTANCIHLAYLAEGYRAEEM---GAFIADARAAMEALF-EHEPF	48
MER310625	-------PFEVLQQASDTTNCIHLAYLAEGYRPEEM---NMFIADARTAMEALF-AHEPY	49
MER310636	-------PYQTILKAKDTTRCIHIAYLAEGYKSDEM---DVFLHDAQKATDAIF-SHEPY	49
MER310634	--YNNRTPFVEMLTPKDSTNCIHIAYIAEGYQTSEM---PTFINDVKNTMEALF-AHEPF	54
MER310649	----------------DTSNCIHIAYIAEGYQPAEM---PTFISDVNNAMEALF-AHEPF	40
MER310618	---NHPTPYRTLQQSADTSHCIHIAFVAEGYRPEEM---DKFVGDARRATDALF-EHEPF	53
MER384772	---NHPTPYRTLQQSADTSHCIHIAFVAEGYRPEEM---DKFVGDARRATDALF-EHEPF	53
MER179368	---NKPTPYVTLQQAADTTRCIHIAYVAEGYTDAEM---PVFLKDAQEATEAIF-AHEPF	53
MER310611	-------PYVTLQKAKDTTRCIHIAYVAEGYREDEM---HTFLNDAKIAMEALF-EHEPF	49
MER310644	-------PYTTLQTAEDTTKCIHIAYVAEGYTAEEM---PIFLEDARIATEALF-EHEPF	49
MER191127	-------PYETLQKAADTTRCIHIAYVAEGYTEAEM---ANFIEDCRTANEALF-AHEPF	49
MER310633	-------PYETLQQAADPSHCIHIAYIAEGYTEAEM---PTFIKDCRIAMEALF-AHEPF	49
MER310643	-------PYETLQQAADPSHCIHIAYIAEGYTETEM---PIFIKDCRIAMEALF-AHEPF	49
MER310624	-------PYETLQQAADTTNCIHIAYVAEGYTEQEM---NTFLTDCRVATEALF-AHEPF	49
MER310628	-------PYETIQPAADPEHCIHIAYVAEGYTESQM---DSFIEDCKTANEALF-AHEPF	49
MER310629	-------PYETLQQAADTSRCIHIAYVAEGYTEAEM---PTFLNDCRTAMEALF-AHEPF	49
MER310619	-------PYTTLQTAADTTRCIHIAYVAEGYTEAEM---PAFINDCRTAMEALF-AHEPF	49
MER310623	-------PYETLQKAADTTRCIHIAYIAEGYTEAEM---PTFLNDCRTAMEALF-AHEPF	49
MER310626	-------PYETLQKAADPTRCIHIAYIAEGYTDAEM---PAFLNDCRTAMEALF-AHEPF	49
MER227814	------VPVKTICKTASPEKAIDIAVIAEGYTAKQR---AKFYKDAQQLADNLF-SHEPF	50
MER310637	-----------VHKAKDNAKAINVVFVAEGFKEEEM---GKFMDAVKVSIEQLL-AHKPF	45
MER310621	-----IPAYTTVHRGDSVNKCVNVVILAEGYTASEM---AKFREHAQITCEQIF-NHSPF	51
MER310646	--------PYRILQRGNVKNPIDVAILAEGYTAHEM---NRFYQDAEKAMESIF-NHEPF	48
MER166667	-----------------------LAIMAEGYTEDEM---ETFYKDSQTACEAIF-AHEPF	33

MER172449	----------YLWQGGSSDKCIDVAIMAEGYAANES---DLFYKDAAVACEALF-AHEPF	46
MER176437	----------YLLQSGTAEDCIDVAILAEGYTQAEM---DLFYKDAEIACEALF-DHEPF	46
MER384774	-------PHQYIQKSGNETDCIDVAIMAEGYTATEM---ELFYKDAGKACESLF-FYEPF	49
MER384777	-------PHKYLLKSGSEADCIDVAILAEGYTEAEM---DLFYKDAEKACESLF-FYEPF	49
MER061231	--TNHITPHRYLLQNGNAADCIDVAIMAEGYTEKEM---DIFYKDAQTACDALF-SHEPF	54
MER173341	--TNHITPHRYLLQSGNTADCIDVAIMAEGYTEKEM---DIFYKDAQTACDALF-SHEPF	54
MER069670	-------PHKYLLKSGNEEQCIDVAILAEGYTTSEM---ETFYKDAAIACEALF-SHEPF	49
MER066231	-------PHRYMLQSGNEKECIDVAILAEGYTEKEM---DLFYQDAQKACESLF-SHEPF	49
MER142057	-------PHRYMLQSGSEKDCIDVAILAEGYTEKEM---DVFYQDAQRTCESLF-SHEPF	49
MER161878	-------PHRYMLQSGNEKDCIDVAILAEGYTEKEM---DVFYQDAQRTCESLF-SYEPF	49
MER172135	-----------------------VAILAEGYTPEEM---PVFYEDAAIACESLF-AHEPF	33
MER172340	----------YLLQNGDADKCIDVAILAEGYTPQEM---QTFYKDAGIACESLF-AHEPF	46
MER310645	-----------------------VAILAEGYTPQEM---QTFYEDAGIACESLF-AHEPF	33
MER310620	----PRYETFEVVYNGNPDHRIDLVLLPEGYSAMEK---EKFMTACKVFAEEFF-SYSPF	52
MER310635	----PRYETFEVAYNGNPSTRVDIVLVPEGYTQNEK---EKFAAACRVFAEEFF-SYSPF	52
MER114395	------------------SDKVDIVIIPDGYTRADS---IKFLKDCERFSNYLF-ASSPF	38
MER384773	---------FKVHYSGDHHKKLDIVFLPEGYTVDEM---EKFRSDCNRFAEYLF-EFSPF	47
MER310632	-------PVRRLQDEGDMAEKVDFVFLPEGYTEAEM---GKFEADARRFMELLF-TIPPY	49
MER310612	--------ITKIQYNGDSSGKVDLVFLAEGYTADEQ---EKFVADAKRFTEALF-KTPPY	48
MER310614	--------ITKIQYNGDSSGKVDLVFLAEGYTADEQ---EKFVADAKRFTEALF-KTPPY	48
MER314302	--------ITKIQYNGDSSGKVDLVFLAEGYTADEQ---EKFVADAKRFTEALF-KTPPY	48
MER191128	--------ITKIRYNGDSSGKVDLVFLAEGYTADEQ---EKFVADAKRFTEALF-KTPPY	48
MER095537	--------ITKIQYNGDSSGKVDLVFLAEGYTADEQ---EKFVADAKRFTEALF-KTPPY	48
MER310613	--------ITKIQYNGDSSGKVDLVFLAEGYTADEQ---EKFVADAKRFTEALF-KTPPY	48
MER191120	--------VHQIQKSGDSTEKVDLVFIAEGYTADEQ---EKFVADANRFTEALF-ATPPF	48
MER191122	--------VHQIQKSGDSAEKVDLVFIAEGYTTDEQ---EKFVADAKRFTEALF-ATPPF	48
MER191124	-------PIKPILQNGSPQKKVDIAVIPEGYTQEQM---DKFVADVKRLFDYLF-ATPPY	49
MER310606	----PQYPIKTILENGSYEKKLDIAVLPEGYTQAEM---DKFLSDAKRLFDYLF-SIAPY	52
MER092644	-------PSTKIYGHKPPQNAVDIAILAEGYTTEEM---SKFKKDAQRFIDYMM-SVEPF	49
MER255292	-------PIKTLLHNGSSSNKVDLAILSEGYTADQM---EKFYADAQRMIDYMF-TISPF	49
MER310630	----------------SPSEKVDLVILAEGYTAAEA---DKFYADAQRMTDYMF-TIPPF	40
MER191121	--TQPSYPIKQLLNNGNAAKKVDIAILAEGYTAAEA---EKFYADAQRMTDYMF-TIPPF	54
MER384768	-------PVKKILYNGEASKKVDIAILAEGYTQAET---EKFYADAQRMTDYMF-TISPF	49
MER065305	-------PVKKILYNGEASKKVDIAILAEGYTQAET---EKFYADAQRMTDYMF-TISPF	49
MER384770	-------PVKKILYNGEASKKVDIAILAEGYTQAET---EKFYADAQRMTDYMF-TISPF	49

MER067561	ATHSSLWNVWLIESISETNILRNNKN-E--------------------------------	77
MER168445	KTYRDYFNVYIIPALSKEKGADNHST-G----------------------------VQKD	86
MER310622	KENAARFNVRAVWAPSDDSGVTIPGE-N----------------------------VWRN	83
MER384775	KENAARFNVRAVWAPSDDSGVTIPGE-N----------------------------VWRN	83
MER384771	NQFKDKINITGIYKASEESGIDEPGA-G----------------------------IFKN	68
MER064266	RSRRRDFNVWGLCPPTEEGGVSRPLT-G----------------------------IHRR	80
MER066009	RSRKADFNVWGLCPPAEEKGVSRPLT-G----------------------------VHRR	80
MER118905	RSRKADFNVWGICPPAEEKGVSRPLT-G----------------------------VHRR	80
MER162996	KERANDFNVWALAVPTQESGVSRPST-G----------------------------VHHA	82
MER065708	KEHRQDFNVWGLCPPAAESGISRPST-G----------------------------IHHR	87
MER141845	KEHKARFNVWGLVPASAQSGISRPST-G----------------------------LHRR	87
MER068550	KERKSDFNVWGLVPAAAQSGISRPST-G----------------------------VHRR	80
MER251522	KERKSDFNVWGLVPAAAQSGISRPST-G----------------------------VHRR	80
MER191119	RSMADRFNVYALCTASVDGYGG-TST-FFAATAKG------------------------G	89
MER310605	RSMADRFNVYALCTASVDGYGG-TST-FFAATAKG------------------------G	89
MER310604	RSYADRFNVYALCTASESTFDNGGST-FFDVIVDKYNSP------------------VIS	91
MER191125	RSYADRFNVYALCTASESTFDNGGST-FFDVIVDKHNSP------------------VIS	96
MER016067	RSYADRFNVYALCTASESTFDNGGST-FFDVIVDKYNSP------------------VIS	96
MER105722	RSYADRFNVYALCTASESTFDNGGST-FFDVIVDKYNSP------------------VIS	96
MER105702	RSYADRFNVYALCTASESTFDNGGST-FFDVIVDKYNSP------------------VIS	96
MER191126	REYSDRINIYAVQAVSNEPGIGVYGG-K-----------------------------SPD	84
MER013883	RTYRNYLNVYAVQTPSAESGVDCDPA-R--------------------------EDQERD	81
MER062296	KSYRNYLNVYALEIPSPQSGVDCDPG-L--------------------------SSPKVD	81
MER220607	KSYRSYFNVYAVSIASPESGVDCDPG-L--------------------------AAPRRD	87
MER193549	KSYRGLFNVWRVDIPSPVSGVSGDPA-R--------------------------D-VVRD	78
MER019878	KSYEGLFNVWAVKAVSEDSGVTGDPT-R--------------------------D-VVRN	80
MER310615	KSYEGLLNVWAVKAVSEDSGVTGDPT-R--------------------------D-VVRN	80
MER289439	TTYRNLFNVWTVDAVSNESGVSGDPD-R--------------------------A-TVRD	72
MER179396	TTYQNLFNVWTVDAVSNDSGVTGDPD-R--------------------------G-AVRD	72
MER310638	TTYRDLFNVWTVDAVSAQSGVSGDPG-P--------------------------D-TVRD	65
MER384778	ASYRGLFNVWAVDAVSHDSGVSGDPT-K--------------------------D-VVRD	65
MER310639	ASYKELFNVWSVDAVSNQSGVSGDPG-K--------------------------D-VVKD	80
MER310648	ASYKDLFNVWTVDAVSNQSGVSGDPS-K--------------------------D-VVKD	80
MER098980	NKYKSFINVWLVNVISNESGVDNDPS-P--------------------------G-VNRD	80

```
MER131394      KSYKDQFNVWMVKVVSKESGVDHDPQ----------------------------G-TLRD      68
MER310627      KSYKNSFNVWAVNVVSQQSGVDNDPR----------------------------G-TYRN      79
MER163974      NEFKDTIKIYALGVVSNESGAKADTA-INQEQA---------------------NADTRD      81
MER310631      DEYADAVKIYAIGVCSNESGVRADRA-RTQAEA---------------------DADTRD      77
MER058697      KEYKNHFNVWRVEVPSNQSGAGNGSP--------------------------------ID      76
MER166461      SDYRDYFNVWLVEKPSQQSGIGIGSP--------------------------------LA      68
MER133451      NDYRNYFNFFAIRTPSKESGTSNPGA-APDAYPD-------------------QPVGMKD      88
MER059337      ------------------------------------------------------------      0
MER191123      RQYTSYFNVFAIRTPSEESGAIHKGV-SNDCPKEDHGHVNITDRFNKYTRNTNVPTSNPK      107
MER173069      ------------------------------------------------------------      0
MER177104      ------------------------------------------------------------      0
MER203593      KQNQLKFNMIAVMPPSVESGTSEPNK-G----------------------------IWKN      80
MER310647      QSSRTKFSCVAVEAPSEESGVSIPDK-G----------------------------IWKK      80
MER310616      KSSRSRFNIVAVKSPSAESGASNPGK-G----------------------------IWKN      79
MER310625      KSMRQWFNIVAVKSPSMESGASEPGK-G----------------------------VWKN      80
MER310636      RSMKDKFNIIAVEAPSEESGTSIPSR-G----------------------------IWKN      80
MER310634      KSMKDRFSIVAVEAPSKDSGTSEPSK-G----------------------------IWKN      85
MER310649      KSMKDRFSVVAVEAPSKDSGTSEPSK-G----------------------------IWKN      71
MER310618      KATRDRFNVVAVMSPSEESGPSEPSK-G----------------------------IWKN      84
MER384772      KATRDRFNVVAVMSPSEESGPSEPSK-G----------------------------IWKN      84
MER179368      KSMRDRFNIVAVKSPSKQSGPSIPAQ-G----------------------------IWHE      84
MER310611      KTLRDKFNIIAVKSPSIDSGTSEPSR-G----------------------------IWKN      80
MER310644      KTKRNRFNIIAVKSVSMDTGTSIPSQ-G----------------------------VWKK      80
MER191127      KSLRQRFNVVAVKSPSMESGTSEPSK-G----------------------------IWKN      80
MER310633      KSLRSRFNIVAVKAPSVDSGTSEPSK-G----------------------------IWKN      80
MER310643      KSLRSRFNIVAVKAPSVDSGTSEPSK-G----------------------------IWKN      80
MER310624      KSMRNRFNIVAVKSPSEESGTSEPSH-G----------------------------VWKN      80
MER310628      KTLRSKFNIIAVKSPSHDSGTSEPSK-G----------------------------IWKN      80
MER310629      KTLRNRFNVVAVKAPSAESGTSNPGK-G----------------------------IWKN      80
MER310619      KSLRNRFNVVAVKSPSAESGTSNPGK-G----------------------------IWKN      80
MER310623      KALRNRFNVIAVKSPSAESGTSNPGK-G----------------------------IWKN      80
MER310626      KSLRNRFNVVAVKSPSAESGTSIPGK-G----------------------------IWKN      80
MER227814      LKYKSRINIYAIAALSVDTGITKPQA-A----------------------------NWRN      81
MER310637      DKFADNFNFIAVKSASKDSGVSVPRQ-G----------------------------DWKK      76
MER310621      NQLKDKFNFYAVETISEDSGVSVPRE-N----------------------------NWSR      82
MER310646      KSNKEKFNFIAVGSISKDTGVSIPHK-D----------------------------RWRA      79
MER166667      KHLKERFNVVAVATPSHDSGVSIPRK-R----------------------------KWKN      64
MER172449      KKLKDRFNIVAVFSESKDSGVSIPRK-D----------------------------EWKK      77
MER176437      KHLKDRFNIVAVASPSQDSGVSVPRR-D----------------------------EWKS      77
MER384774      KSMRHKFNIIAVASLSEDSGVSVPRK-N----------------------------DWKH      80
MER384777      KSMKSRFNVVAVASPSKDSGVSIPRK-N----------------------------DWKQ      80
MER061231      KKLKDKFNIVAVASPSEDSGVSIPGQ-G----------------------------KWKS      85
MER173341      KKLKEKFNIVAVASPSEDSGVSIPGQ-G----------------------------KWKS      85
MER069670      QSMKNRFNIVAVASPSADSGVSAPKQ-G----------------------------AWKH      80
MER066231      RSMKNKFNIVAVASPSIDSGVSVPRE-N----------------------------QWKH      80
MER142057      RSMKQKFNIVAVASPSTDSGVSVPRA-D----------------------------QWKQ      80
MER161878      RSMKSKFNIVAVASPSTDSGVSVPRE-N----------------------------QWKQ      80
MER172135      RSMKKHFNIVAVASPSEDSGVSVPRL-G----------------------------EWKR      64
MER172340      KSMKKRFNIVAVASPSTDSGVSVPRL-N----------------------------EWKH      77
MER310645      KSMKERFNIVAVASPSIDSGVSVPRL-N----------------------------EWKH      64
MER310620      KENAQKFNIRAVWAPSEETGVTIPGE-H----------------------------IWRN      83
MER310635      KENTARFNIRAVWSPSMESGVTIPGE-H----------------------------VWRN      83
MER114395      KENKDKFNIHAVLAWSEDSGTDIPAE-N----------------------------IWKS      69
MER384773      DELKERINIWGIEAPSGETGADIPAE-N----------------------------IWKN      78
MER310632      DARRSDFNVWAVDVPSEESGTDLSGK-G----------------------------IYRN      80
MER310612      DTRREDFNVWAVDAVSEESGTDVSGK-G----------------------------IFKN      79
MER310614      DTRREDFNVWAVDAVSEESGTDVSGK-G----------------------------IFKN      79
MER314302      DTRREDFNVWAVDAVSEESGTDVSGK-G----------------------------IFKN      79
MER191128      DTRREDFNVWAVDAVSEESGTDVSGK-G----------------------------IFKN      79
MER095537      DTRREDFNIWAVDAVSEESGTDVSGK-G----------------------------IFKN      79
MER310613      DTRREDFNVWAVDAVSEESGTDVSGK-G----------------------------IFKN      79
MER191120      TTRRNDFNVWAVCLVSEESGTDVSGK-G----------------------------IFKN      79
MER191122      TTRRGDFNVWAVCLISEESGTDVSGK-G----------------------------IFKN      79
MER191124      NKHKKDFNIQAILAPSQESGTDMEGSPT----------------------------TYKN      81
MER310606      DKYKKNFNIYAILSPSQESGTDMEGSPT----------------------------NYKN      84
MER092644      KSNRNHFNVYTIESPSQESGTDISGE-N----------------------------IYKN      80
MER255292      DDLKDNFNVYAIGTASYESGTDIPGE-N----------------------------IYKK      80
MER310630      DKLKKDFNVRAIAVPSAESGTDVPGK-N----------------------------IYRN      71
MER191121      DKLKKDFNVYAIAVPSKESGTDVPGK-H----------------------------IYKN      85
MER384768      DKLKNNFNVYAIAVPSQESGTDIPGK-N----------------------------IFKN      80
MER065305      DKLKNNFNVYAIAVPSQESGTDVPGK-N----------------------------IFKN      80
```

MER384770	DKLKNNFNVYAIAVPSQESGTDVPGK-N----------------------------IFKN	80
MER067561	LFLASD-------LSEACTS-PF--SYCPNVQAAYN--------------------QMTA	107
MER168445	TYFNSG--------WRD----DYSDM---GANENTVSTFLETYC-P---------DIVNG	121
MER310622	TACGAS-------FYTF----DSERYQMVTDFQRLRD-----------------M----A	111
MER384775	TACGAS-------FYTF----DSERYQMVTDFQRLRD-----------------M----A	111
MER384771	NLLGTT-------FYSL----GSERYVLTEDNKAMRD-----------------L----A	96
MER064266	SRVGAT-------YDAF----GSERYVLSFENRSLRD-----------------V----A	108
MER066009	TRVGAT-------YDAF----GSERYLLTFENRSMRD-----------------V----A	108
MER118905	TRVGAT-------YDAF----GSERYLLTFENRSMRD-----------------V----A	108
MER162996	SALGTR-------YDIF----GSERYVLTLDNRALRD-----------------I----A	110
MER065708	SPLGTT-------YDTF----DSERYILTTENRAMRD-----------------A----A	115
MER141845	SPVGAT-------YDAF----GSERYVLTFDNEAFRD-----------------T----A	115
MER068550	SPIGAT-------YDAF----GSERYVLTFDNKAFRE-----------------A----A	108
MER251522	SPIGAT-------YDAF----GSERYVLTFDNKAFRE-----------------A----A	108
MER191119	ISTNKG-------NWRN---HVLERIIGPAFIEKIHDAHIPNETHPNENTMDHNYRQYDY	139
MER310605	ISTNKG-------NWRN---HVLERIIGPAFIEKIHDAHIPNETHPNENTMDHNYRQYDY	139
MER310604	NNLHGS-------QWKN---HIFERCIGPEFIEKIHDAHIKKKCDPNTIPSGSEYEPYYY	141
MER191125	NNLHGS-------QWKN---HIFERCIGPEFIEKIHDAHIKKKCDPNTIPSGSEYEPYYY	146
MER016067	NNLHGS-------QWKN---HIFERCIGPEFIEKIHDAHIKKKCDPNTIPSGSEYEPYYY	146
MER105722	NNLHGS-------QWKN---HIFERCIGPEFIEKIHDAHIKKKCDPNTIPSGSEYEPYYY	146
MER105702	NNLHGS-------QWKN---HIFERCIGPEFIEKIHDAHIKKKCDPNTIPSGSEYEPYYY	146
MER191126	TYFHVKVYGKAAGFTNG----GDERAKALRTELEEN-----------------YLDEGAN	123
MER013883	TALDMG-------FFNGCDPEALERLLTVDEEAAEA-----------------AADLVPG	117
MER062296	TPLQMG-------FWGGCNPASVQRLLTVSATAAAQ-----------------YADLVAG	117
MER220607	TPLDMG-------FWGGCNPQSVQRLLTVDDAAAQR-----------------YADLVPG	123
MER193549	TPLGMT-------FWCD----GLERLLCVDEDRAKA-----------------YAALAPG	110
MER019878	TALGSY-------FWCG----DTERLICVDLDRTKA-----------------YADRAPA	112
MER310615	TALGSY-------FWCG----DTERLICVDLDRTKA-----------------YADRAPA	112
MER289439	TALGSY-------FWCG----DIERLLCVDEAKVDG-----------------YVAKAPE	104
MER179396	TALGSY-------FWCE----DIERLLCVDQPKVDA-----------------YVAKAPA	104
MER310638	TALGSY-------FWCE----SIERLLCVDQPKVDA-----------------YVAKAPA	97
MER384778	TALNSA-------FFCE----GTERLLCVDTDKVES-----------------YAAKAPA	97
MER310639	TALKSY-------FWCD----DIERLLCVDTAKVES-----------------YAAKAPD	112
MER310648	TALGSY-------FWCD----DIERLLCVDTAKVES-----------------YAAKAPE	112
MER098980	TALDMY-------LWCN----NIERLLCLNEAKARQ-----------------YANQAPG	112
MER131394	TALDME-------FWCG----DTERLLCVNTTKAKQ-----------------YAAEAPD	100
MER310627	TALDMT-------FWCG----GTERLLCVNENKALQ-----------------FARLAPE	111
MER163974	TYFGSS-------FWSG----GMQRLLTISSDGSKK-----------------AKQLSDQ	113
MER310631	SYFHAS-------FWTF----GMQRLVEIGEEDKGK-----------------IRDLYRK	109
MER058697	TKFGAH-------FNCY----NIER-LLCVDEGKVL-----------------NYLKSVM	107
MER166461	GSAFKS-------YFSV----SSSRLLLLDDRFSSL-----------------NEAKSVL	100
MER133451	TFFGVS-------FGY-----SIHRLVEVSKFDVLY-----------------G--LMAS	117
MER059337	-----------------------------------VK-----------------KFANESL	9
MER191123	TIFGSS-------FDNM----GLHRLVIPHHEEAIQ-----------------KVL--KD	137
MER173069	--	0
MER177104	--	0
MER203593	TPLGSH-------FDTF----YSERYLTTLHLKKLHD---------------------VL	108
MER310647	TALLSS-------YDTF----YSDRYLTTLHLKKLHD---------------------WL	108
MER310616	TALHSH-------WYTN----YSERYLTTSRLKDMHD---------------------LL	107
MER310625	TALRSH-------WDTN----YSERYLTTSHLKDMHD---------------------LL	108
MER310636	TALQSH-------FDTF----YSARYLTTLRLKKVHD---------------------WL	108
MER310634	TALSSH-------FDTF----YSDRYLTTLNLKDLHD---------------------WL	113
MER310649	TALSSH-------FDTF----YSDRYLTTLNIKDLHD---------------------WL	99
MER310618	TALHSN-------FDTF----YSNRYLTTLHLKDLHD---------------------KL	112
MER384772	TALHSN-------FDTF----YSNRYLTTLHLKDLHD---------------------KL	112
MER179368	TALSSH-------FDTF----YSDRYLTTLHLKDLHN---------------------WL	112
MER310611	TALHSH-------FDTF----YSDRYLTTLSINDLHD---------------------IM	108
MER310644	TVLGSH-------FDTF----YSNRYLTTLNQKNLHD---------------------AL	108
MER191127	TALHSH-------FDTF----YSDRYLTTLHLKELHD---------------------WL	108
MER310633	TALHSH-------FDTF----YSDRYLTTLHLKDLHD---------------------WL	108
MER310643	TALHSH-------FDTF----YSDRYLTTLHLKDLHN---------------------WL	108
MER310624	TALHSH-------FDTF----YSDRYLTTLHLKDLHN---------------------WL	108
MER310628	TALHSN-------FDTF----YSDRYLTTLHLKDLHN---------------------WL	108
MER310629	TALHSN-------FNTF----YSDRYLTTLHLKDLHN---------------------WL	108
MER310619	TALHSN-------FNTF----YSDRYLTTLHLKTLHN---------------------WL	108
MER310623	TALHSN-------FNTF----YSDRYLTTLHLKTLHN---------------------WL	108
MER310626	TALHSN-------FDTF----YSDRYLTTLHLKTLHN---------------------WL	108
MER227814	TALGSH-------YYTF----YSDRYLTTPNVFTVRD---------------------YA	109
MER310637	TAVESN-------FDTF----YSERYLTTNRIKKLHD---------------------VL	104

```
MER310621     TAFSSH-------FDTF----YSDRYLTTSHVKDIHD---------------------AI     110
MER310646     TAFGAH-------FDTF----YSERYLTTRNVKSIHD---------------------AL     107
MER166667     TAVSSH-------FDTF----YSDRYLTTRSVKAMHN---------------------WL     92
MER172449     TAVSSH-------FDTF----YSDRYLTTRSVKAIHN---------------------WL     105
MER176437     TAVSSH-------FDTF----YSDRYLTTRSVKAIHN---------------------WL     105
MER384774     TAFGSH-------FDTF----YSDRYLTTCRVKSIHN---------------------AL     108
MER384777     TAFNSH-------FDTF----YSDRYLTTSRVKSIHN---------------------VL     108
MER061231     TAVSSH-------FNTF----YSDRYLTTSRVKSIHN---------------------WL     113
MER173341     TAVSSH-------FNTF----YSDRYLTTSRVKSIHN---------------------WL     113
MER069670     TAFGSH-------FDTF----YSDRYLTTSRVKAIND---------------------AL     108
MER066231     TAVHSH-------FDTF----YSDRYLTTSRVKAIHN---------------------AL     108
MER142057     TAVHSH-------FDTF----YSERYLTTSRVKSIHN---------------------AL     108
MER161878     TAVHSH-------FDTF----YSDRYLTTSRVKSVHN---------------------AL     108
MER172135     TAFSSH-------FSTF----YSDRYLTTSRVKSIHD---------------------AL     92
MER172340     TAFSSH-------FSTF----YSDRYLTTSRVRAVHD---------------------AL     105
MER310645     TAFGSH-------FSTF----YSDRYLTTSRVKTIHD---------------------AL     92
MER310620     TALKAS-------FYTF----DSERYQMIEDFQNVRD---------------------VA     111
MER310635     TAAQAR-------YYTF----DSERYQMIEDFQGLRD---------------------IA     111
MER114395     TIANSS-------FYTF----DVDRYLMIYDNKILRH---------------------LA     97
MER384773     TLLKSR-------FYTF----DSERYLMTEDYYTVRD---------------------VA     106
MER310632     TAFGSG-------FYTF----GLDRYLTTSDMKSIRD---------------------AL     108
MER310612     TALNSG-------YYTF----GVDRYLTTPDMKSIRD---------------------AV     107
MER310614     TALNSG-------YYTF----GVDRYLTTPDMKSIRD---------------------AV     107
MER314302     TALNSG-------YYTF----GVDRYLTTPDMKSIRD---------------------AV     107
MER191128     TALNSG-------YYTF----GVDRYLTTPDMKSIRD---------------------AV     107
MER095537     TALNSG-------YYTF----GVDRYLTTPDMKSIRD---------------------AV     107
MER310613     TALNSG-------YYTF----GVDRYLTTPDMKSIRD---------------------AV     107
MER191120     TALNSG-------YYTF----GVDRYLTTPDMKSIRD---------------------AV     107
MER191122     TALNSG-------YYTF----GVDRYLTTPDMKSIRD---------------------AV     107
MER191124     TLLDSH-------FYSF----GMDRYLTCPSLFKAAD---------------------VA     109
MER310606     TLFDSH-------FYSF----GLDRYLTCPSLFKVAD---------------------VA     112
MER092644     TILNSH-------FYTF----GEPRYLTTLSSFKISD---------------------IA     108
MER255292     TVFDAS-------FYTF----DMQRYLTANNFKQIAD---------------------AA     108
MER310630     TAFNSS-------FYTF----NQERYLTTNSLKEIAD---------------------VA     99
MER191121     TAFNAS-------FYTF----NQERYLTTNSMQAIAD---------------------AA     113
MER384768     TAFNSS-------FYTF----NQERYLTTNSICEIAD---------------------AA     108
MER065305     TAFNSS-------FYTF----NQERYLTTNSICEIAD---------------------AA     108
MER384770     TAFNSS-------FYTF----NQERYLTTNSICEIAD---------------------AA     108

MER067561     AVPHADTGYVQLNLQYII--GVAKYLGDFG-------------------IT--------Y     138
MER168445     SVTVD-------DVPVCLLVNDSRYGGICWSWSSGKAYTIVPTTEGLWQYSSDEELGVSI     174
MER310622     AHVPY-------DYIYVL-SNTQKYGGGGIF----------------NFYGIS----AAH     143
MER384775     AHVPY-------DYIYVL-SNTQKYGGGGIF----------------NFYGIS----AAH     143
MER384771     ALVPY-------DAIYIM-CNTERYGGGGIY----------------NFYCTF----TSD     128
MER064266     SFAPY-------DAVAIL-VNAETYGGGGIF----------------NLYATV----AAD     140
MER066009     SFAPY-------DALAIL-ANGETYGGGGIF----------------NLYATV----AAD     140
MER118905     SFAPY-------DALAIL-ANGETYGGGGIF----------------NLYATV----AAD     140
MER162996     QYAPY-------EFIEIL-VNNDTYGGGGIF----------------GQFSTA----AAN     142
MER065708     SFAPY-------EFVEIL-VNGKTYGGGGIF----------------NLYGTV----AID     147
MER141845     AFAPY-------DFVEIL-VNGNTYGGGGIF----------------GLYGTV----ASD     147
MER068550     AFAPY-------EFVEIL-VNGNTYGGGGIF----------------GLYGTV----ASD     140
MER251522     AFAPY-------EFVEIL-VNGNTYGGGGIF----------------GLYGTV----ASD     140
MER191119     VYENI-------NQFVVL-ANSGEYFGGSHD----------NKQYGIHYI-VA----SAR     176
MER310605     VYENI-------NQFVVL-ANSGEYFGGSHD----------NKQYGIHYI-VA----SAR     176
MER310604     VHDYI-------AQFAMV-VNTKSDFGGAYN----------NREYGFHYF-IS----PSD     178
MER191125     VHDYI-------AQFAMV-VNTKSDFGGAYN----------NREYGFHYF-IS----PSD     183
MER016067     VHDYI-------AQFAMV-VNTKSDFGGAYN----------NREYGFHYF-IS----PSD     183
MER105722     VHDYI-------AQFAMV-VNTKSDFGGAYN----------NREYGFHYF-IS----PSD     183
MER105702     VHDYI-------AQFAMV-VNTKSDFGGAYN----------NREYGFHYF-IS----PSD     183
MER191126     V-----------GTIHILCNDTGSYGASVNP------------L---FSFSTN----S-E     152
MER013883     VSADN-------RQILAL-ANSGTYGGRGGA------------------TATA----SSG     147
MER062296     TSSAN-------RQILAI-GNSDTYGGAGGS------------------YATA----SGG     147
MER220607     TSAAN-------RQILAI-GNSSTYGGAGGT------------------YATA----SGG     153
MER193549     V-----------DQIAAM-ANSAKYGGAGYTD---------------EEMATF----SGG     139
MER019878     T-----------DLVIVV-SNSAKYGGAGYSGL--------AADYGYDGVSTL----SSD     148
MER310615     T-----------DLVIVV-SNSAKYGGAGYSGL--------AADYGYDGVSTL----SSD     148
MER289439     A-----------DLVLVL-ANSAKYGGAGYNQP--------SPTLGYEGIATA----SAG     140
MER179396     A-----------DLVIVL-ANSAKYGGAGYNEP--------SATLGYEGISTA----SAG     140
MER310638     A-----------DLVIVL-ANSAKYGGAGYNEP--------SATLGYEGISTA----SAG     133
MER384778     A-----------DLVVEL-SNSTKYGGAGYNDI--------SSQVGYDGIATA----SSD     133
```

```
MER310639      A-----------DLVVVL-SNSTKYGGAGYT-L--------TSQVGYDGIATA----SSD      147
MER310648      A-----------DLVVVL-GNSTKYGGAGYT-L--------TSQVGYDGIATA----SSD      147
MER098980      V-----------DAIVAV-GNTAKYGGAGYR-----------------DLSTV----SGA      139
MER131394      V-----------DQVLAL-GNSTKYGGAGYPS---------------EDVGTS----SGG      129
MER310627      V-----------DQVVAL-ANTTKYGGAGG------------------RVATS----SGG      137
MER163974      YLPAA-------DFNVVI-VNATTYGGSGG------------------DVCVA----S-L      142
MER310631      YLPDT-------DYAIVM-VNSEVYGGSGG------------------EISIV----S-R      138
MER058697      PANAM-------DKVLVV-VNTEKYGGAGG------------------QVATM----S-L      136
MER166461      PESAT-------EIFLVV-SNTEKYGGAGYA-----------------NVATM----S-T      130
MER133451      HFPAY-------DLIVVL-ANTDYYGGSGG------------------QIAVH----T-L      146
MER059337      GFGGY-------DQVLVI-GNTKEYGGAGYD-----------------GIGTL----T-M      39
MER191123      HIPNY-------SQVVIL-VNSPYYGGSGG------------------KYATA----T-V      166
MER173069      -------------QVLCI-VNSSKYGGSGG------------------MIATC----S-T      23
MER177104      -------------QVLCI-VNSTKYGGSGG------------------SVATA----S-V      23
MER203593      AGIPY-------EHIIVL-VNTDRYGGGGIY----------------NSYNLT----YAH      140
MER310647      AGVPY-------EHIIVL-VNTNRYGGGGIL----------------NSYDLC----AAH      140
MER310616      AGTPY-------EHILVL-VNSDGYGGGGIL----------------NSYILS----TTR      139
MER310625      AGTPY-------EHILVL-VNSDRYGGGGIL----------------NSYILS----TMH      140
MER310636      AGTPY-------EHIIIL-VNTPEYGGGGIL----------------NSYNLA----MTH      140
MER310634      AGIPY-------EHIIVL-VNTNHYGGGGIL----------------NSYNLS----MAH      145
MER310649      SGIPY-------EHIIVL-VNTDHYGGGGIL----------------NSYNLS----MAH      131
MER310618      AGTPY-------EHIIVL-VNTAEYGGGGIL----------------NSYNLS----MTD      144
MER384772      AGTPY-------EHIIVL-VNTAEYGGGGIL----------------NSYNLS----MTD      144
MER179368      AGTPY-------EHIIVL-VNSDKYGGGGIL----------------NSYNLT----TCH      144
MER310611      AGTPY-------EHLIVL-VNTDKYGGGGIL----------------NSYNLS----MTH      140
MER310644      AGTPY-------EHIIVL-VNTSEYGGGGIL----------------NSYNLS----MTH      140
MER191127      AGTPY-------EHIIVL-VNTEKYGGGGIL----------------NSYNLS----MVR      140
MER310633      AGTPY-------EHIIVL-VNTEKYGGGGIL----------------NSYNLS----MAH      140
MER310643      AGTPY-------EHIIVL-VNTEKYGGGGIL----------------NSYNLS----MAH      140
MER310624      AGTPY-------EHIIVL-VNTKEYGGGGIL----------------NSYNLS----MTH      140
MER310628      AGTPY-------EHIIVL-VNTDKYGGGGIL----------------NSYNLS----MTK      140
MER310629      AGTPY-------EHIIVL-VNTDNYGGGGIL----------------NSYNLS----MTH      140
MER310619      AGTPY-------EHIIIL-VNTENYGGGGIL----------------NSYNLS----MVR      140
MER310623      AGTPY-------EHIIVL-VNTENYGGGGIL----------------NSYNLS----MVR      140
MER310626      AGTPY-------EHIIVL-VNTENYGGGGIL----------------NSYNLS----MVR      140
MER227814      SLVPY-------DAIYIL-ANTKAYGGGGIY----------------NFYTLA----SAD      141
MER310637      AGIPY-------EHIIIL-ANTDVYGGGGIY----------------NSYTLT----TTG      136
MER310621      AGIPY-------AHIIIL-ANTDVYGGGGIF----------------NAYTLT----TTG      142
MER310646      AGIPY-------EHIILL-ANTDQYGGGGIY----------------NSYTLT----SAH      139
MER166667      AGIPY-------EHIIIL-ANTDTYGGGGIY----------------NSYLLT----TAH      124
MER172449      AGIPY-------EHIIIL-ANTDTYGGGGIY----------------NSYTLT----TAH      137
MER176437      AGIPY-------EHIIIL-ANTDTYGGGGIY----------------NSYTLT----TTH      137
MER384774      AGIPY-------EHIIII-ANTAEYGGGGIY----------------NSYTLT----AAH      140
MER384777      SGIPY-------EHIIII-VNTEEYGGGGIY----------------NSYTLT----AAH      140
MER061231      AGIPY-------EHIIIL-ANTDTYGGGGIY----------------NSYTLT----TAH      145
MER173341      AGIPY-------EHIIIL-ANTDTYGGGGIY----------------NSYTLT----TAH      145
MER069670      AGIPY-------EHIIIL-ANTEQYGGGGIY----------------NAFTLT----TAH      140
MER066231      AGIPY-------EHIIIL-ANTDVYGGGGIY----------------NSYTLT----TAH      140
MER142057      AGIPY-------EHIIIL-ANTDVYGGGGIY----------------NSYTLT----TAH      140
MER161878      AGIPY-------EHIIIL-ANTDVYGGGGIY----------------NSYTLT----TAH      140
MER172135      AGIPY-------EHIIIL-ANTEEYGGGGIY----------------NSYTLT----TAH      124
MER172340      AGIPY-------EHIIIL-ANTEEYGGGGIY----------------NSYTLT----TAH      137
MER310645      AGIPY-------EHIIIL-ANTKEYGGGGIY----------------NSYTLT----TAH      124
MER310620      AHVPY-------DYIYIL-SNTQKYGGGGIY----------------NFYGIS----AAN      143
MER310635      AHAPY-------DHIYVL-SNTQKYGGGGIY----------------NFYGIS----AAH      143
MER114395      SNAPY-------DQIYVL-VNTSKYGGGSIY----------------NHYSVC----VSD      129
MER384773      SNAPY-------DQIFII-VNTAKYGGGAIY----------------NFYNVT----ASD      138
MER310632      WNVPC-------DAIFIL-VNSSDYGGGGIY----------------NYYAMG----TAD      140
MER310612      WNAPC-------DAIFIL-VNTDAYGGGGMY----------------NFYAMG----TAD      139
MER310614      WNAPC-------DAIFIL-VNTDAYGGGGMY----------------NFYAMG----TAD      139
MER314302      WNAPC-------DAIFIL-VNTDAYGGGGMY----------------NFYAMG----TAD      139
MER191128      WNAPC-------DAIFIL-VNTDAYGGGGMY----------------NFYAMG----TAD      139
MER095537      WNAPC-------DAIFIL-VNTDAYGGGGMY----------------NFYAMG----TAD      139
MER310613      WNAPC-------DAIFIL-VNTDAYGGGGMY----------------NFYAMG----TAD      139
MER191120      WNVPC-------DAIFLL-INTDMYGGGGMY----------------NFYACG----TAD      139
MER191122      WNVPC-------DAIFLL-INTDMYGGGGMY----------------NFYACG----TAD      139
MER191124      AAVPY-------DQLFVV-VNTKEYGGGAFY----------------NVINLN----VSD      141
MER310606      SGVPY-------DQLFVI-VNTKEYGGGAFY----------------NLINLN----VSD      144
MER092644      ANVPY-------DQIFVI-VNTPRYGGGGFY----------------NVINLV----SAD      140
MER255292      SLVPY-------DQIYVL-VNTEMYGGGAFY----------------NHLNLT----SVD      140
MER310630      AAVPY-------DQLYVL-VNTPIYGGGGFY----------------NHLNLG----TAD      131
```

```
MER191121      SAVPY-------DQIYVL-VNTSTYGGGAFY----------------NHLNLA----SAD      145
MER384768      SVVPY-------DQLYIL-VNTETYGGGGFY----------------NHLNLG----TAD      140
MER065305      SVVPY-------DQLYIL-VNTETYGGGGFY----------------NHLNLG----TAD      140
MER384770      SVVPY-------DQLYIL-VNTETYGGGGFY----------------NHLNLG----TAD      140
                                :  .     .

MER067561      T-SSTSKISVHEL-GHSHAWLKDEYASPE----------GISGYGINVS----SHCFPPN      182
MER168445      G-DWK-NTFLHEYGGHAFGRLADEY-----------------------------------      197
MER310622      HPTRTGKIHVHEF-GHLLLGLGDEYVG-T-TSYDDMYAKSIEPWEPNLT----TLVGFGD      196
MER384775      HPTRTGKIHVHEF-GHLLLGLGDEYVG-T-TSYDDMYAKSIEPWEPNLT----TLVGFGD      196
MER384771      N-QFSDYIFLHEF-GHSFGGLADEYYTSS-VAYNEFYPQGIEPVEPNIT----RLLDKDN      181
MER064266      N-RWAGYVFVHEL-GHSLAALADEYFTSE-TAYLSS-EARPEPWEPNVT----A--NPRA      190
MER066009      N-RWAGYIFVHEL-GHHLAALADEYFTSD-TAYLSP-EGRPEPWEPNVT----A--DPKA      190
MER118905      N-RWAGYIFVHEL-GHHLAALADEYFTSD-TAYLSP-EGRPEPWEPNVT----A--DPKA      190
MER162996      N-DWADYLFVHEF-GHHFAGLADEYYTSP-VAYQSS-GARMEPWEPNVT----ALRDPAR      194
MER065708      N-AWANYVGVHEF-GHHFAGLADEYYTSD-VAYNSE-TKRKEPWEPNVT----ALLDPAN      199
MER141845      S-LWAPYIFVHEF-GHHFAGLADEYYTSD-SAYLPS-EDRAEPWEKNVT----ALKDPSQ      199
MER068550      S-LWAPYVFVHEF-GHHFAGLADEYYTSE-SVYAPA-ADRLEPWEKNVT----ALHSPEG      192
MER251522      S-LWAPYVFVHEF-GHHFAGLADEYYTSE-SVYAPA-ADRLEPWEKNVT----ALHSPED      192
MER191119      N-AYSAFTQRHEL-GHGLFHLGDEYNYST-VPVDE----WNYTTSLNMT----ATKDPTK      225
MER310605      N-AYSAFTQRHEL-GHGLFHLGDEYNYST-VPVDE----WNYTTSLNMT----ATKDPTK      225
MER310604      S-YRASKTFAHEF-GHGLLGLGDEYSNGY-LLDD------KELKSLNLS----SVEDPEK      225
MER191125      S-YRASKTFAHEF-GHGLLGLGDEYSDGY-LLDD------KELKSLNLS----SVEDPEK      230
MER016067      S-YRASKTFAHEF-GHGLLGLGDEYSNGY-LLDD------KELKSLNLS----SVEDPEK      230
MER105722      S-YRASKTFAHEF-GHGLLGLGDEYSNGY-LLDD------KELKSLNLS----SVEDPEK      230
MER105702      S-YRASKTFAHEF-GHGLLGLGDEYSNGY-LLDD------KELKSLNLS----SVEDPEK      230
MER191126      D-NSDGTAMAHEI-AHSIGRLGDEYERYT--------------NKPNTS----DTANPDT      192
MER013883      N-ALSALISPHEL-GHSLGELQDEYPYYF-RDTSL-------------------------      179
MER062296      N-ALSALITPHEL-GHSLGGLQDEYDYYA-RGERGAPYVGGEPSSIHHTVLTEQEMLAQQ      204
MER220607      N-ALSALISPHEL-GHSLGGLDDEY-----------------------------------      176
MER193549      N-ERAGEVLPHEL-GHSLGDLADEYDYYA-YPGDGSRYDGPEFSEVNVSVRDAGRMRAER      196
MER019878      N-DRSTLIAAHEM-AHSIGLLADEYQYEG----YG-DYPYPEPAHPNASTLTADEMARQR      201
MER310615      N-DRSTLIAAHEM-AHSIGLLADEYQYEG----YG-DYPYPEPAHPNASTLTADEMARQR      201
MER289439      N-EKSGQVAIHET-GHSLGKLADEYFYPG-YPGYE-RYTGAEPADSNISTLTADEIGRQR      196
MER179396      N-AKSGQVAIHET-GHSLGKLADEY-----------------------------------      163
MER310638      H-PKSGQVAIHET-GHSLGKLADEYFFFL-RPRLR-EVHRHRARRPQ-------HLRPRR      182
MER384778      N-DRSDQIAVHET-GHSLGKLADEYAYGE----SG-TYSGPEPAQANLSTLSAAQMTAQR      186
MER310639      H-ADSDQVAVHET-GHSLGKLADEY-----------------------------------      170
MER310648      H-ADSDQIAVHET-GHSLGKLADEYFYPE----YG-TYTGAEPDEGNATKLTADQMTAQQ      200
MER098980      N-VLSGQIALHEL-GHSVGGLADEYYTPG------TTYSGGELLAPN------ATTDSTG      185
MER131394      N-EQAGQVVVHEF-GHSIGDLADEYTYGG-----GDTYTGPEVGEVNASIKDRATQESEK      182
MER310627      N-TAAGQIVLHEL-GHTIGGLADEYDYPD------DRYTGGEPREVNASIYASAQMQQKR      189
MER163974      N-NESLEMMLHEL-GHTTAKLSDEYFAGA------SYA----AEMPNMT----AESDPAK      186
MER310631      N-DESLEMLLHEL-GHTIGILSDEYFAGN------SYA----GEYVNMS----AESDPKK      182
MER058697      A-PQAIDLALHEL-GHSFAKLADEYDYGT------CQVH--EPDNANAT------ANSSG      180
MER166461      H-SSAVGLALHEV-GHTFGKLADEYSYGS------CNLNS-EPPEKNVS------MLTSN      175
MER133451      N-KDANTIGVHEI-GHTFGRLSDEYWAGS------LYG----SETANMT----ANSDPAT      190
MER059337      H-KQSVELALHEF-GHSAYHLADEYDYGN------CVIEGDENEKANVT----KETDRNK      87
MER191123      N-AASNDIAVHEI-GHSFAVLADEYWAGN------QYA----IESPNRS----QEGNPTK      210
MER173069      H-AQAAQIAIHEM-GHSAFGLADEYGGNG------AGTPAGEPSQPNVT----RDTSRTT      71
MER177104      N-ASASQIAIHEM-GHSAFGLADEYGGNG------SGTPAGEPLQPNVT----RDTNRAT      71
MER203593      GKH-FRPVVVHEF-GHSFGGLGDEYPYGD--D-DPMYFADTEPWEPNLT----TKHDFNG      191
MER310647      HPT-FRPVVVHEF-GHSFAGLADEYAYDT-EE-VPMYPHDVEPWEKNIT----TKVNFKG      192
MER310616      NAW-AKPVVVHEF-GHSFAGLADEYAYDA-EP-VDLYPLDVEPWEKNIT----TRVDFRD      191
MER310625      NQW-SKPVVVHEF-GHSFAGLADEYAYEG-DE-VNMYPLDVEPWEKNIT----TKVDFHG      192
MER310636      HPS-FKPVVVHEF-GHSFAGLADEYAYDF-ES-IDMYPTDVEPWEPNIT----TKVNFDE      192
MER310634      HRL-TRPVIVHEF-GHSFAGLADEYAYEA-EQ-IPMYPHDIEPWEENIT----TLVDFKS      197
MER310649      HRL-TRPVVVHEF-GHSFAGLADEYGYEY-EQ-IPMYPHDIEPWEPNIT----TLVNFHG      183
MER310618      NPW-FRPVVVHEF-GHSFAGLGDEYAYEA-EQ-IPMYPHNVEPWEPNLT----TLADFKG      196
MER384772      NPW-FRPVVVHEF-GHSFAGLGDEYAYEA-EQ-IPMYPHNVEPWEPNLT----TLADFKG      196
MER179368      QKW-FKPVVVHEF-GHSFAGLADEYAYEQ-EQ-IPMYPHDVEPWEKNIT----TLADFHG      196
MER310611      HPA-YRSVVVHEF-GHSFAGLGDEYAYEQ-EQ-IPMYPHDIEPWEPNLT----TLHNFHN      192
MER310644      HPL-YKQVVVHEF-GHSFAGLADEYA----------------------------------      164
MER191127      NPY-FKPVVVHEF-GHSFAGLGDEYAYEK-EQ-INMYPTDVEPWEPNLT----TLVDFHG      192
MER310633      HPQ-FKPVVVHEF-GHSFAGLGDEYAYAK-EE-INMYPKDVEPWEPNLT----TLVDFHN      192
MER310643      HAY-FKPVVVHEF-GHSFAGLGDEYAYAK-EE-INMYPKDVEPWEPNLT----TLVDFHN      192
MER310624      NKY-FKPVVVHEF-GHSFAGLADEYAYEQ-ES-LNMYPTDIEPWEANIT----TMVNFDN      192
MER310628      HSL-FKPVVVHEF-GHSFAGLGDEYAYGY-EQ-IPMYPHDIEPWEPNLT----TLVNFDS      192
MER310629      HRA-FKPVVVHEF-GHSIGDLADEYAYDY-EA-IPMFPHDIEPWEKNIT----TMVDFKS      192
MER310619      HPA-FKPVVVHEF-GHSFAGLGDEYG--Y-GD-IPMYPKDIEPWEPNLT----TLVDLKS      190
MER310623      HSA-FKPVVVHEF-GHSFAGLGDEYG--Y-DD-IPMYPHDIEPWEANLT----TLVDFKS      190
```

```
MER310626      HPA-FKPVVVHEF-GHSFAGLGDEYG--Y-GD-MPMYPHDIEPWEPNLT----TLVDLKS       190
MER227814      SKRAQAEVTVHEF-GHSFAGLADEYFYDN-DALNGMYNLKNEPWEPNIT----TLVQFGA       195
MER310637      HKD-FKPVVVHEF-GHSFAGLADEYFYPN-DVLSDLISNQTEPWEQNIT----TLVDFDS       189
MER310621      HAA-FKPVVVHEF-GHSFAGLADEYFYES-DVLDNTYLHSVEPWEPNIT----TLVDFTL       195
MER310646      HEM-FEPVVVHEF-GHSFGGLADEYFYEQ-DTMTDIYPLDIEPWEPNIT----TLVNFES       192
MER166667      HPM-FKPVVVHEL-GHSFGGLGDEYAYDT-AP-SPQYPYSVEPWEPNIT----TLVDFES       176
MER172449      HSM-FRPVVVHEF-GHSFGGLADEYAYDE-AP-SPLYPYDIEPWEPNIT----TLVHFED       189
MER176437      NPM-FRPVVVHEF-GHSFAGLADEYAYTE-EP-SPLYPYDIEPWEQNIT----TLVNFES       189
MER384774      HPT-FKPVVVHEF-GHSFAGLGDEYFYED-DVMTDTYPLNVEPWEPNIT----TRTDFSS       193
MER384777      HPT-FKPVVVHEF-GHSFGGLGDEYFYEE-DVMTDTYPLDVEPWEPNIS----TRVDFSS       193
MER061231      HPD-FQPVVVHEF-GHSFGGLADEYA----------------------------------       169
MER173341      HPD-FQPVVVHEF-GHSFGGLADEYA----------------------------------       169
MER069670      HPN-FRPVVVHEF-GHSFGGLADEYFYDE-DVMNGLYPLNIEPWEQNIT----TRINFAS       193
MER066231      HPM-FKPVVVHEF-GHSFGGLADEYFYDD-DVMTDTYPLDVEPWEQNIS----TRVNFAL       193
MER142057      HPM-FKPVVVHEF-GHSFGGLADEYFYED-DTMTDTYPLDVEPWEQNIT----TRVNFAS       193
MER161878      HPM-FKPVVVHEF-GHSFGGLADEYFYDN-DVMTDTYPLDVEPWEQNIS----TRVNFAS       193
MER172135      HSM-FRPVVVHEF-GHSFGGLADEYFYDN-DVMTDTYPLDVEPWEQNIS----TRIDFTS       177
MER172340      HAM-FRPVVVHEF-GHSFGGLADEYFYDN-DIMTDTYPLDIEPWEQNIS----TQVDFAS       190
MER310645      HPM-FRPVVVHEF-GHSFGGLADEYFYDN-DVMTDTYPLDVESWEQNIS----TQVNFTS       177
MER310620      HPTRTGKIYVHEF-GHVLLGLGDEYV--GNVSYNDMYPKDVEPWEANLT----TLVDFGR       196
MER310635      HPNRTGKIYVHEF-GHVLLGLGDEYI--GNVSYNDMYPTDVEPWEANLT----TLTDFGR       196
MER114395      NP-NSEYIFVHEF-GHGFAALGDEYY-TSEVAYSEFYPLDVEPLDPNLT----TLVNFDS       182
MER384773      NR-LSKLIFVHEF-GHGLAGLADEYA--DTSTYNEFYNLETEPWEPNIT----TLVDFDS       190
MER310632      HE-RTAKVFVHEL-GHSFAGLADEYF-ESEVAYGNFYNVKLEPWEPNIT----TLVNFDS       193
MER310612      NP-RTPVVFVHEF-GHSFAGLADEYF-SSEVAYQDFYNLKYEPWEPNIT----TLVNFDA       192
MER310614      NP-RTPVVFVHEF-GHSFAGLADEYF-SSEVAYQDFYNLKYEPWEPNIT----TLVNFDA       192
MER314302      NP-RTPVVFVHEF-GHSFAGLADEYF-SSEVAYQDFYNLKYEPWEPNIT----TLVNFDA       192
MER191128      NP-RTPVVFVHEF-GHSFAGLADEYF-SSEVAYQDFYNLKYEPWEPNIT----TLVNFDA       192
MER095537      NP-RTPVVFVHEF-GHSFAGLADEYF-SSEVAYQDFYNLKYEPWEPNIT----TLVNFDA       192
MER310613      NP-RTPVVFVHEF-GHSFAGLADEYF-SSEVAYQDFYNLKYEPWEPNIT----TLVNFDA       192
MER191120      NP-RTPVVFTHEF-GHSFAGLADEYF-SSEVAYQDFYNLKYEPWEPNIT----TLVDFGS       192
MER191122      NP-RTPVVFTHEF-GHSFAGLADEYF-SSEVAYQDFYNLKYEPWEPNIT----TLVDFDS       192
MER191124      NK-YAEKTFVHEF-GHGFVGLADEYYYEGEEGMADRYNLKVEPWEANIT----TLVNFQS       195
MER310606      NK-YAEKTFVHEF-GHGLVGLADEYYYE--EGMGSRYNLKIEPWEANIT----TLVNFQS       196
MER092644      NY-LSEKVFVHEF-GHGFAGLGDEYYNNS-GGTTDLYNLKIEPWEPNLT----TLVDFGK       193
MER255292      HN-LSEKVFVHEF-GHGFVGLADEYY-DSQTAFESMYNPNIEPWEENIT----TLVDFQN       193
MER310630      NE-LSEKVYIHEF-GHGFVGLADEYYYDWDPTFQDMYNQKIEPWEENIT----TLVDFGS       185
MER191121      NE-LSEKVYIHEF-GHGFVGLADEYYYDWDPTFQETYDSKIEPWEENIT----TLVNFES       199
MER384768      NN-LSEKVYIHEF-GHGFVGLADEYYYDWDPTFQEMYNTKIEPWEENIT----SLVNFES       194
MER065305      ND-LSEKVYIHEF-GHGFVGLADEYYYDWDPTFQEMYNTKIEPWEENIT----SLVNFES       194
MER384770      ND-LSEKVYIHEF-GHGFVGLADEYYYDWDPTFQEMYNTKIEPWEENIT----SLVNFES       194
                         **  .*    * ***

MER067561      LSW---------------------------------------------------------       185
MER168445      ------------------------------------------------------------       197
MER310622      KFWSR-------------------------------------------------------       201
MER384775      KFWSR-------------------------------------------------------       201
MER384771      IKWKSLL-----------------------------------------------------       188
MER064266      AKWKDLL-----------------------------------------------------       197
MER066009      AKWADLV-----------------------------------------------------       197
MER118905      AKWADLV-----------------------------------------------------       197
MER162996      LKWKQ-------------------------------------------------------       199
MER065708      LKWKDLV-----------------------------------------------------       206
MER141845      LKWKALL-----------------------------------------------------       206
MER068550      LKWKHLV-----------------------------------------------------       199
MER251522      LKWKHLV-----------------------------------------------------       199
MER191119      VKWKQLLGFR-----------------------NTYTCPHLDYYPYTYNSS-RDCLMRET       261
MER310605      VKWKQLLGFR-----------------------NTYTCPHLDYYPYTYNSS-RDCLMRET       261
MER310604      IKWRQLLGFR-----------------------NTYTCRNA-YGSKMLVSS-YECIMRDT       260
MER191125      IKWRQLLGFR-----------------------NTYTCRNA-YGSKMLVSS-YECIMRDT       265
MER016067      IKWRQLLGFR-----------------------NTYTCRNA-YGSKMLVSS-YECIMRDT       265
MER105722      IKWRQLLGFR-----------------------NTYTCRNA-YGSKMLVSS-YECIMRDT       265
MER105702      IKWRQFLGFR-----------------------NTYTCRNA-YGSKMLVSS-YECIMRDT       265
MER191126      IKWSKMLGFR--------------------GI-GITTA----GTDTAFAPS-RECMMRRL       226
MER013883      ------------------------------------------------------------       179
MER062296      RKWWRWLGEESES----------------GGRIGRY-EAGQYAGSGIWRPS-RHSMMKVL       246
MER220607      ------------------------------------------------------------       176
MER193549      AKWWYWLG----------------------------------------------------       204
MER019878      TKWASWLG----------------------------------------------------       209
MER310615      TKWASWLG----------------------------------------------------       209
MER289439      VKWHRWLG----------------------------------------------------       204
```

```
MER179396      ------------------------------------------------------------      163
MER310638      ------------------------------------------------------------      182
MER384778      QKWYRWMG----------------------------------------------------      194
MER310639      ------------------------------------------------------------      170
MER310648      KKWYRWIG----------------------------------------------------      208
MER098980      QKWASYLGRSTPD----------------GGVIGAY-QGGEQYEYGIYRPS-QDSLMRSL      227
MER131394      VKWHQWMGEETPD----------------GGTIDTY-DGCRYYEHGIFRPS-DNSIMREL      224
MER310627      TKWYQYLG----------------------------------------------------      197
MER163974      VRWSRFIGKN--------------------GV-GVYEYDNG--GNGWYRPH-QNCKMRFL      222
MER310631      VRWSRFIGKN--------------------GI-GVYEYDNG--GDGWYKPH-QNCKMRYL      218
MER058697      AKWRHWMDVD--------------------SNVGVF-EGAMYCTRGMYRPT-QNSMMKEL      218
MER166461      VKWSHWMG----------------------------------------------------      183
MER133451      IRWKNWLDNP--------------------QI-GIHKHGPEGDAAHWHKPANGTCLMEYL      229
MER059337      IKWRHLI-----------------------------------------------------      94
MER191123      VRWKNWIGTN--------------------GI-GVYAYGSKASPSTWFRPH-EFCKMQYL      248
MER173069      NKWRDLI-----------------------------------------------------      78
MER177104      NKWRTLI-----------------------------------------------------      78
MER203593      -KWENLIKD---------------------KKAGFI-EGGGYLSKGVWRGY-ENCRMRTN      227
MER310647      -KWENLIGKD--------------------PKAGFY-EGAGYSPKGVYRAY-PDCRMRTN      229
MER310616      -KWENLLGKD--------------------KAAGFY-EGAGYKLKGVYRAY-EDCRMRTN      228
MER310625      -KWENLIGRD--------------------KKAGLY-EGAGYKLKGVFRGY-RDCRMRTN      229
MER310636      -KWKDLIGKN--------------------SAIGLF-EGAGYAIKGVYRPA-LHCRMRDN      229
MER310634      -KWNDLIKKNTLIPTPTTPA--------NKNKVGVY-EGAGYSVKGVYRPM-QDCRMRTN      246
MER310649      -KWEDLIQKKTPIPTPPTAA--------NRHHVGVY-EGAGYALKGVYRPT-MDCRMRTN      232
MER310618      -KWENLIDKKTPVPTPATGKY--------KNKVGVY-EGAGYSLKGVYRGM-QDCRMRTN      245
MER384772      -KWENLIDKKTPVPTPATGKY--------KNKVGVY-EGAGYSLKGVYRGM-QDCRMRTN      245
MER179368      -KWENMIDKKTKIPTPLSKKE-----KEAVSKVGVF-EGAGYSLKGVYRGV-QDCRMRIN      248
MER310611      -KWEKMIDKGTPIPTPPTKNL---------QKIGVF-EGAGYSLKGVYRGQ-QDCRMRTN      240
MER310644      ------------------------------------------------------------      164
MER191127      -KWENLINKKTPIPTPQPADLDKPNARYDKWKVGVY-EPAGYSQHGVYRAY-PDCRMRTN      249
MER310633      -KWEGMIDKKTPLPTPEPTDLDKPNARRDKWKVGAY-EPAGYAQHGVYRAY-PDCRMRTN      249
MER310643      -KWEGMIDKKTPLPTPEPTDLDKPNARRDKWKVGAY-EPAGYAQHGVYRAY-PDCRMRTN      249
MER310624      -KWKKMVKKGTPLPTPQPTDLDTPCANASKWKVGAY-EPAGYSQHGVYRAF-PDCRMRTN      249
MER310628      -KWKDMVKKGTKIPTT----FNETEKKMEKWNIGAY-EPAGYSMHGVYRPF-PDCRMRTN      245
MER310629      -KWSDMVKKGTKIPTEQPADLDKPNAKKEKWQIGAY-EPAGYSMHGVYRAY-PDCRMRTN      249
MER310619      -KWGDLI-----------------------------------------------------      196
MER310623      -KWSDMV-----------------------------------------------------      196
MER310626      -KWADMVTKNTPLPTPQPADLDKPNAKQDKWKIGAY-EPAGYSKHGVYRAF-PDCRMRTN      247
MER227814      -KWKKDLPQGTPIPTPPTEEN--------KAKIGVF-EGGGYLSKGIYRPY-FDCRMRTN      244
MER310637      -KWKKMLKKGTPVPTLEKDSK--------KYPLGVY-EG--LEGRKVYKGT-LDCRMKTN      236
MER310621      -KWQDMLVPQTPVPTNTDLAD--------KFSVGVF-EGAGYSAKGIYRPA-IDCRMKTN      244
MER310646      -KWKSMLKEGTPIPTSSSDTK--------SYPLGVY-EGGGYSTKGIYRPA-FDCRMRTN      241
MER166667      -KWKDMLPAQTPVPTPAETDP-----NTIYTKVGVY-EGGGYTLKGIYRPT-TECRMKIN      228
MER172449      -KWKDML-----------------------------------------------------      195
MER176437      -KWKDML-----------------------------------------------------      195
MER384774      -KWEDMLPAATPVPTPQQASG--------KYPIGVY-EGGGYSAKGIYRPS-FDCRMRTN      242
MER384777      -KWKDMLPAGVPVPTPPQMNK--------QYPVGVY-EGGGYSAKGIYRPS-FDCRMRTN      242
MER061231      ------------------------------------------------------------      169
MER173341      ------------------------------------------------------------      169
MER069670      -KWEDMLTKTTPVPTPVADKA--------KYPIGVY-EGGGYSAKGIYRPA-FDCRMRTN      242
MER066231      -KWEDMLAPNTPVPTPVAQHQ--------NYPVGVY-EGGGYSAKGIYRPA-FNCRMKTN      242
MER142057      -KWEDMLPPHIPVPTPVAQQK--------NYPVGVY-EGGGYSFKGIYRPA-YNCRMKTN      242
MER161878      -KWKDMLPSGAPIPTPIAEKK--------KYPVGVY-EGGGYSAKGIYRPA-YDCRMKTN      242
MER172135      -KWKDMLAQGTPVPTPSSESG--------TYPVGVY-EGAGYSAKGIYRPA-DNCRMRTN      226
MER172340      -KWKDMLPSNIPIPTPISVSE--------KYPTGVY-EGGGYSAKGIFRPA-DNCRMRTN      239
MER310645      -KWKDMLAKDTPIPTPTGNSE--------KYPVGVY-EGGGYSAKGIYRPA-DDCRMRTN      226
MER310620      KDWKHLL-----------------------------------------------------      203
MER310635      KEWKKML-----------------------------------------------------      203
MER114395      -KWKDLIEEGTPVPTPNTKEY--------RDKIGVF-EGGGYSAKGVYRPA-FDCTMKSI      231
MER384773      -KWKSLIDENTPVPTPEDSTY--------ADKIGVF-EGGGYVLKGVYRPT-VNSLMRSF      239
MER310632      -KWKDLLSEKTPVPTPVAEN---------EARPGVY-EGGGYVAKGVYRPM-VHCMMRDY      241
MER310612      -KWKDLLPANTPVPTPLDDAH--------KDKAGVF-EGGGYIAKGIYRPM-DHCMMRDY      241
MER310614      -KWKDLLPANTPVPTPLDDAH--------KDKAGVF-EGGGYIAKGIYRPM-DHCMMRDY      241
MER314302      -KWKDLLPANTPVPTPLDDAH--------KDKAGVF-EGGGYIAKGIYRPM-DHCMMRDY      241
MER191128      -KWKDLLPTNTPIPTPLDDAH--------KDKAGVF-EGGGYIAKGIYRPM-DHCMMRDY      241
MER095537      -KWKDLLPTNTPIPTPLDDAH--------KDKAGVF-EGGGYIAKGIYRPM-DHCMMRDY      241
MER310613      -KWKDLLPTNTPIPTPLDDAH--------KDKAGVF-EGGGYIAKGIYRPM-DHCMMRDY      241
MER191120      -KWKDLLPADTPIPTPLDAGH--------KDKAGVF-EGGGYLSKGIYRPM-DHCMMRDY      241
MER191122      -KWKDLLPANTPIPTPLDAEH--------KDKAGVF-EGGGYLSKGIYRPM-DHCMMRDY      241
MER191124      -KWKDLVEKQTPIPTPRTEQY--------KNKVGAF-EGGGYLFKGMYSPM-EDCRMKSI      244
MER310606      -KWKDLVDKKTPIPTPRTEKY--------KDKVGAF-EGGGYLSKGMYSPM-QDCRMKSI      245
```

```
MER092644      -KWKHKVNKNTPIPTPRTELY--------KNSIGVF-EGGGYTPKGIYSPV-QDCRMKSN      242
MER255292      -KW---------------------------------------------------------      195
MER310630      -KWKDMVQKNTPIPTPRTKKY--------QKVVGAF-EGGGYTSKGVYSPM-QDCRMKSN      234
MER191121      -KWKDMVKKGTPIPTPRTSKY--------KNTLGAF-EGGGYTSKGVYSPM-QDCRMKSN      248
MER384768      -KWKDMVKKKTPIPTPRTPKY--------KDVVGAF-EGGGYTSKGIYSPA-QDCRMKSN      243
MER065305      -KWKDMVKKKTPIPTPRTPKY--------KDVVGAF-EGGGYTSKGIYSPA-QDCRMKSN      243
MER384770      -KWKDMVKKKTPIPTPRTPKY--------KNVVGAF-EGGGYTSKGIYSPA-QDCRMKSN      243


MER067561      -----------------------      185
MER168445      -----------------------      197
MER310622      -----------------------      201
MER384775      -----------------------      201
MER384771      -----------------------      188
MER064266      -----------------------      197
MER066009      -----------------------      197
MER118905      -----------------------      197
MER162996      -----------------------      199
MER065708      -----------------------      206
MER141845      -----------------------      206
MER068550      -----------------------      199
MER251522      -----------------------      199
MER191119      F-QNDFCDVCKLQGIKVM-----      278
MER310605      F-QNDFCDVCKLQGIKVM-----      278
MER310604      --NYQFCEVCRLQGFKRMSQLVK      281
MER191125      --NYQFCEVCRLQGFKRM-----      281
MER016067      --NYQFCEVCRLQGFKRM-----      281
MER105722      --NYQFCEVCRLQGFKRM-----      281
MER105702      --NYQFCEVCRLQGFKRM-----      281
MER191126      GQ--PFCEVCKMELARKL-----      242
MER013883      -----------------------      179
MER062296      GY--YFDQVSRERMTQRI-----      262
MER220607      -----------------------      176
MER193549      -----------------------      204
MER019878      -----------------------      209
MER310615      -----------------------      209
MER289439      -----------------------      204
MER179396      -----------------------      163
MER310638      -----------------------      182
MER384778      -----------------------      194
MER310639      -----------------------      170
MER310648      -----------------------      208
MER098980      NK--PF-----------------      231
MER131394      GR--EF-----------------      228
MER310627      -----------------------      197
MER163974      GKQYAFCEVCKEQ----------      235
MER310631      GRQFPFCEVCK------------      229
MER058697      GQ--PFYAVNESEIVRRI-----      234
MER166461      -----------------------      183
MER133451      NQ--ELCAVCNEATIERL-----      245
MER059337      -----------------------      94
MER191123      VA--PFCNVCQ------------      257
MER173069      -----------------------      78
MER177104      -----------------------      78
MER203593      E-EPEFCLVCQQALQRL------      243
MER310647      E-NPEFCPACKQALQG-------      244
MER310616      E-TPEFCPACR------------      238
MER310625      E-IPEFCPACR------------      239
MER310636      E-TPDFCPVCQ------------      239
MER310634      E-NPEFCDVCR------------      256
MER310649      E-NPEFCPACQ------------      242
MER310618      Q-YPEFCPVCQQAIHRL------      261
MER384772      Q-YPEFCPVCQQAIHRL------      261
MER179368      E-TPEFCAVCK------------      258
MER310611      E-YPEFCAVCK------------      250
MER310644      -----------------------      164
MER191127      M-HPEFCPACNLGLTKL------      265
MER310633      A-HPEFCPAC-------------      258
MER310643      A-HPEFCPACTQAITRL------      265
MER310624      V-KPDFCPVCQ------------      259
MER310628      Q-NPEFCPVCR------------      255
```

```
MER310629      N-NPEFCPVCQ------------      259
MER310619      -----------------------      196
MER310623      -----------------------      196
MER310626      Q-NPNFCPVCQ------------      257
MER227814      T-AKGFCPVC-------------      253
MER310637      Q-YPEFCPVCQ------------      246
MER310621      T-CKDFCPVCQ------------      254
MER310646      T-FANFCPACQ------------      251
MER166667      E-APRFCPVC-------------      237
MER172449      -----------------------      195
MER176437      -----------------------      195
MER384774      E-YPEFCPVCQRAIRRI------      258
MER384777      E-YPTFCPVCQRSIRRI------      258
MER061231      -----------------------      169
MER173341      -----------------------      169
MER069670      E-YPTFCPVCQ------------      252
MER066231      E-YPEFCPVCQ------------      252
MER142057      E-HPEFCPVCQRAIRRI------      258
MER161878      E-YPEFCPVCQRAIRRM------      258
MER172135      E-YPTFCPVCQ------------      236
MER172340      E-YPEFCPVCQRALRRI------      255
MER310645      E-YPTFCPVCQRALRRI------      242
MER310620      -----------------------      203
MER310635      -----------------------      203
MER114395      S-IDNFCAVCK------------      241
MER384773      S-SHEFNEVCR------------      249
MER310632      H---PFCPVCR------------      249
MER310612      A---PFCPAC-------------      248
MER310614      A---PFCPAC-------------      248
MER314302      A---PFCPAC-------------      248
MER191128      A---PFCPAC-------------      248
MER095537      A---PFCPAC-------------      248
MER310613      A---PFCPAC-------------      248
MER191120      A---PFCPAC-------------      248
MER191122      A---PFCPAC-------------      248
MER191124      Q-SNEFCPVCSRAIERM------      260
MER310606      Q-SNEFCPVC-------------      254
MER092644      T-PENFCPVC-------------      251
MER255292      -----------------------      195
MER310630      E-PKGFCPVC-------------      243
MER191121      D-PKGFCPVCERAIRRI------      264
MER384768      E-PKGFCPVC-------------      252
MER065305      E-PKGFCPVC-------------      252
MER384770      E-PKGFCPVC-------------      252
```

Fig. S6. Alignment of AK183_Peptidase_M64 sequences from Merops. Species from fecal collections were shown as the highlight.

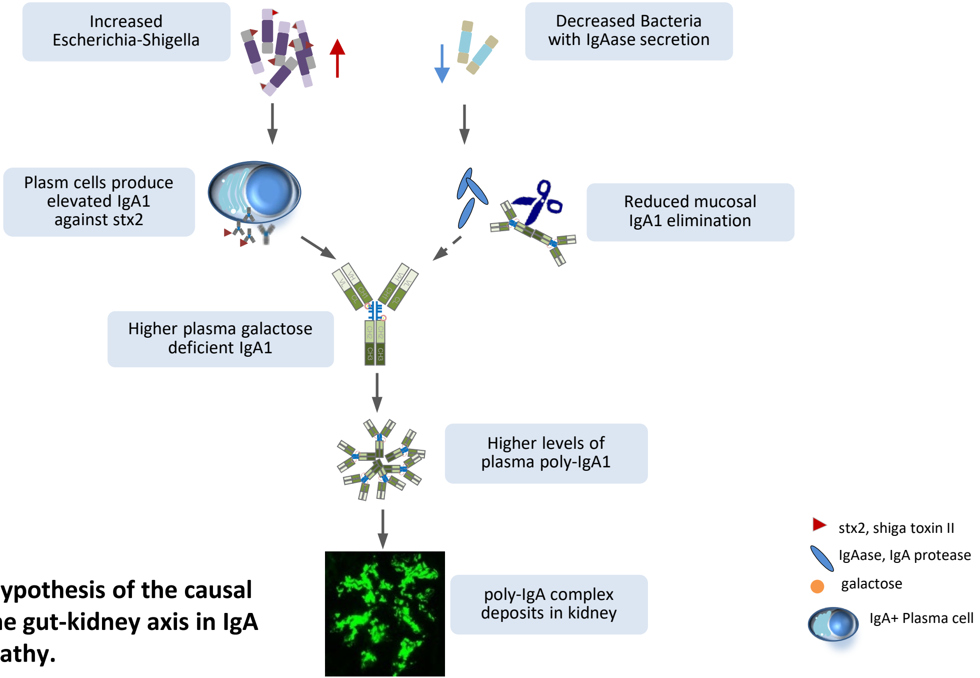

Fig. S7. Hypothesis of the causal role of the gut-kidney axis in IgA Nephropathy.
